# The clinical value and potential molecular mechanism of the downregulation of MAOA in hepatocellular carcinoma tissues

**DOI:** 10.1002/cam4.3434

**Published:** 2020-09-15

**Authors:** Yu‐Yan Pang, Jian‐Di Li, Li Gao, Xia Yang, Yi‐Wu Dang, Ze‐Feng Lai, Li‐Min Liu, Jie Yang, Hua‐Yu Wu, Rong‐Quan He, Zhi‐Guang Huang, Dan‐Dan Xiong, Li‐Hua Yang, Lin Shi, Wei‐Jia Mo, Deng Tang, Hui‐Ping Lu, Gang Chen

**Affiliations:** ^1^ Department of Pathology First Affiliated Hospital of Guangxi Medical University Nanning Guangxi Zhuang Autonomous Region P.R. China; ^2^ School of Pharmacy Guangxi Medical University Nanning Guangxi Zhuang Autonomous Region P.R. China; ^3^ Department of Cell Biology and Genetics School of Pre‐Clinical Medicine Guangxi Medical University Nanning Guangxi Zhuang Autonomous Region P.R. China; ^4^ Department of Medical Oncology First Affiliated Hospital of Guangxi Medical University Nanning Guangxi Zhuang Autonomous Region P.R. China; ^5^ Department of Pathology Second Affiliated Hospital of Guangxi Medical University Nanning P.R. China

**Keywords:** gene chip, HCC, immunohistochemical staining, *MAOA*, RNA‐seq, tissue microarray

## Abstract

**Background:**

Hepatocellular carcinoma (HCC) remains one of the most common cancers worldwide and tends to be detected at an advanced stage. More effective biomarkers for HCC screening and prognosis assessment are needed and the mechanisms of HCC require further exploration. The role of *MAOA* in HCC has not been intensively investigated.

**Methods:**

In‐house tissue microarrays, genechips, and RNAsequencing datasets were integrated to explore the expression status and the clinical value of *MAOA* in HCC. Immunohistochemical staining was utilized to determine MAOA protein expression. Intersection genes of *MAOA* related co‐expressed genes and differentially expressed genes were obtained to perform functional enrichment analyses. In vivo experiment was conducted to study the impact of traditional Chinese medicine nitidine chloride (NC) on *MAOA* in HCC.

**Results:**

*MAOA* was downregulated and possessed an excellent discriminatory capability in HCC patients. Decreased *MAOA* correlated with poor prognosis in HCC patients. Downregulated MAOA protein was relevant to an advanced TNM stage in HCC patients. Co‐expressed genes that positively related to *MAOA* were clustered in chemical carcinogenesis, where CYP2E1 was identified as the hub gene. In vivo experiment showed that nitidine chloride significantly upregulated *MAOA* in a nude mouse HCC model.

**Conclusions:**

A decreased *MAOA* level is not only correlated with aggressive behaviors in males but also serves as a promising biomarker for the diagnosis and prognosis of HCC patients. Moreover, *MAOA* may play a role in AFB1 toxic transformation through its synergistic action with co‐expressed genes, especially CYP3A4. *MAOA* also serves as a potential therapy target of NC in HCC patients.

## Graphical abstract

A decreased MAOA level is not only correlated with aggressive behaviors in males but also serves as a promising biomarker for the diagnosis and prognosis of HCC patients. Moreover, MAOA may play a role in AFB1 toxic transformation through its synergistic action with co‐expressed genes, especially CYP3A4. MAOA also serves as a potential therapy target of traditional Chinese medicine nitidine chloride in hepatocellular carcinoma patients.

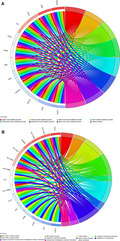

## 1 INTRODUCTION

Hepatocellular carcinoma (HCC) threatens the lives of humans worldwide, and it is regarded as one of the most dominant causes of cancer‐related deaths.^1^ Surgery, namely liver transplantation and hepatectomy, is currently the most effective approach to treat HCC patients.^2^ Despite the major progress made in treating HCC, the prognostic status of HCC patients remains poor because of the lack of significant symptoms until advanced cancer in most HCC patients, thus causing a poor prognosis and a heavy burden on these patients.^3^, ^4^ To date, many protein markers, such as AFP and PIVKA‐II, have been found to screen for HCC patients.^5^ There are also a lot of promising biomarkers for HCC therapy or prognosis evaluation.^6^, ^7^ Nonetheless, according to the 2018 global cancer statistics, 841 080 new cases and 781 631 deaths of liver carcinoma were reported.^8^ Moreover, the incidence of liver cancer is increasing rapidly compared with other forms of cancer based on American cancer statistics in 2020.^9^ Therefore, the situation confronting HCC patients remains grim. Substantial efforts have been made to analyze the carcinogenesis and deterioration mechanisms of HCC.^10^, ^11^, ^12^ Accumulating research supports that HCC risk is associated with inter‐individual genetic variation.^13^ Moreover, HCC correlates with chronic hepatitis B or C virus infection, aflatoxin, and alcohol abuse.^8^, ^14^ Despite the expended time and efforts, the precise molecular mechanisms of HCC remain obscure. Deeper insight into the complex molecular mechanisms of HCC is urgently needed. Furthermore, it is important to discover more novel target genes relevant to the pathogenesis and progression of HCC and to provide potential approaches for early diagnosis and effective treatment regimens of HCC.^15^

Monoamine oxidase‐A (MAOA), an enzyme distributed in the central nervous system and peripheral tissues and organs, has a profound function of breaking down monoamine substances, namely dopamine, epinephrine, and norepinephrine.^16^ Previous studies have documented that the *MAOA* gene, also known as the warrior gene, is relevant to abnormal behaviors (ie antisocial, aggressive, criminal, etc) in males.^17^ Conversely, lower *MAOA* expression is associated with greater happiness of females after adjusting for possible impact of other factors, namely ages, incomes, and interpersonal relationship.^18^ The multiple effects of the *MAOA* gene have attracted the interest of researchers.

Recently, the overexpression of *MAOA* has been reported in various cancers, such as prostate cancer,^19^, ^20^ renal cell carcinoma,^21^ classical Hodgkin lymphomas,^22^ and glioma.^23^ Conversely, other studies have found a downregulation trend of *MAOA* in several cancers.^24^, ^25^ *MAOA* was also found to participate in the formation and progression of tumors. According to Jason et al, *MAOA* induced the distal metastasis of prostate cancer.^26^ *MAOA* was also proved to be correlated with the worse prognosis of HER‐2 subtype breast cancer.^27^ Further studies supported that upregulated *MAOA* promoted angiogenesis in breast cancer ^28^ and contributed to the transition from epithelial to mesenchymal in lung cancer.^29^ Li et al discovered that *MAOA* was downregulated in cholangiocarcinoma (ICC) specimens through hypermethylation orinterleukin‐6 (IL‐6) signaling and that *MAOA* could serve as a prognostic biomarker for ICC patients.^30^ However, only one study has mentioned the expression significance of *MAOA* in HCC to date.^31^ They found that *MAOA* hampers HCC metastasis by inhibiting the epinephrine system, which mediates the metastasis of tumor cells by binding to a specific receptor and transactivating the EGFR signal pathway.^31^ The role of *MAOA* in the carcinogenesis and progression of HCC has not been intensively studied, and the mechanisms underlying *MAOA* in HCC remain obscure.

In the present study, we evaluated the *MAOA* expression levels in HCC patients compared with non‐cancer groups by integrating in‐house tissue microarray data and datasets downloaded from Gene Expression Omnibus (GEO), Array Express, Sequence Read Archive (SRA), Oncomine, the Cancer Genome Atlas (TCGA), and the Genotype‐Tissue Expression project (GTEx). According to the *MAOA* expression value, a diagnostic trial was performed to estimate the clinical value of *MAOA* in HCC screening. The prognostic capacity of *MAOA* in HCC patients was also investigated. We also shed light on the clinical significance of the MAOA protein and determined the potential molecular mechanisms of *MAOA* in HCC. Finally, we addressed the effect of traditional Chinese medicine (TCM) nitidine chloride (NC) on the *MAOA* expression to provide potential therapy for HCC patients.

## 2 MATERIALS AND METHODS

### 2.1 Datasets downloaded from GEO, array express, SRA, oncomine, TCGA, and GTEx databases

HCC datasets were downloaded from the GEO database (https://www.ncbi.nlm.nih.gov/geo/), SRA (https://www.ncbi.nlm.nih.gov/sra), Oncomine (https://www.oncomine.org/resource/main.html), and ArrayExpress (https://www.ebi.ac.uk/arrayexpress/), and RNA sequencing (RNA‐seq) data were downloaded from TCGA (https://cancergenome.nih.gov/) and GTEx (https://gtexportal.org/home/) using the following MESH terms: (malignancy OR cancer OR tumor OR neoplasm OR neoplasia OR carcinoma OR carcinomatosis) AND (hepatocellular OR liver OR hepatic OR HCC). HCC datasets meeting the following criteria were included in the analysis: (a) the specimens were collected from humans, (b) both HCC sample tissues and non‐cancer liver tissues were provided, (c) the expression profile could be downloaded, and (d) the expression level of *MAOA* is available. If the datasets were duplicated, only the latest version would be preserved. Several studies were excluded because of the following reasons: (a) datasets of cell lines, (b) sample size <3, and (c) lack of *MAOA*.

### 2.2 Statistical analyses and the clinical significance of *MAOA* in HCC patients

Independent‐samples *t*‐test was performed to compare the expression status of *MAOA* between the HCC and non‐cancer groups using GraphPad Prism v8.0 software. To assess the capacity of *MAOA* to distinguish HCC patients from people without liver cancer, the receiver operating characteristic (ROC) curve was plotted using GraphPad v8.0 software. The area under the curve (AUC)> 0.70 was recognized to have moderate diagnostic capability, and AUC >0.90 indicated great diagnostic capability. Subsequently, the standard mean difference (SMD) was computed to assess the general expression level of *MAOA* in HCC patients using STATA v12.0 software. To evaluate the heterogeneity of the included datasets, we performed a heterogeneity test in STATA v12.0, where *I* ^2^ >0.50 indicated significant heterogeneity, and a random effect analysis was required. A Begg's funnel plot was drawn to determine whether publication bias occurred, and a *P*‐value ≥.05 indicated no publication bias. A summary of the receiver operating characteristic (sROC) curve was presented, and the specificity and sensitivity of *MAOA* were computed in STATA v12.0. A Kaplan‐Meier curve was plotted to obtain further insight into the association between the *MAOA* expression level and the overall survival condition of HCC patients.

### 2.3 Genetic alteration and mutation types of *MAOA* in HCC

The genetic alteration status of *MAOA* in HCC was explored in the cBioPortal for Cancer Genomics (https://www.cbioportal.org/) database,^32^ which provides an interactive interface to visualize the results. The study on liver hepatocellular carcinoma (TCGA, Provisional) consisting of 440 samples was queried for our analysis. The mutation types of *MAOA* in HCC were interpreted in the Catalogue of Somatic Mutations in Cancer (https://cancer.sanger.ac.uk/cosmic).^33^

### 2.4 Identification of co‐expressed genes and differentially expressed genes of *MAOA*

The Pearson's correlation coefficient of *MAOA* and other detected genes in each expression matrices was computed using R software v3.6.1. The genes meeting the following criteria were identified as significantly co‐expressed genes (CEGs) of *MAOA*: (a) the absolute value of Pearson's correlation coefficient >0.3 and (b) a *P*‐value <.05. The reshape2 and ggplot2 packages were utilized to plot the heatmap of the CEGs of *MAOA* in the datasets using R software v3.6.1. Moreover, a relationship network diagram was plotted with Cytoscape v3.6.1. The Limma Voom package was used to identify differentially expressed genes (DEGs). The standards of DEGs identification were as follows: (a) the absolute value of log_2_FoldChange >1 and (b) an adjusted *P*‐value <.05. The intersection genes of the *MAOA* positively related CEGs and downregulated DEGs and those of the *MAOA* negatively related CEGs and upregulated DEGs were obtained for further analyses.

### 2.5 Disease Ontology, Gene Ontology, and Kyoto Encyclopedia of Genes and Genomes enrichment analyses and the protein‐to‐protein interaction network

The two types of intersection genes acquired were used to undertake the Disease Ontology (DO) (http://disease‐ontology.org/), Gene Ontology (GO) (http://geneontology.org/), and Kyoto Encyclopedia of Genes and Genomes (KEGG) (https://www.genome.jp/kegg/) enrichment analyses respectively. The results were visualized with the aid of DOSE, GO.db, topGO, GSEABase, GOplot, and stringr packages in R v3.6.1 software. The protein‐to‐protein interaction (PPI) network was constructed in STRING (https://string‐db.org/), and the hub genes were detected using Cytoscape v3.6.1 to explore the potential mechanism of *MAOA* underlying HCC.

### 2.6 Evaluation of the clinical value of the MAOA protein expression in HCC patients by immunohistochemical (IHC) staining

A total of 315 HCC and 192 control tissue specimens (ie adjacent normal tissues or non‐HCC tissues) were collected and fixed with formalin from patients in First Affiliated Hospital of Guangxi Medical University, Nanning, Guangxi Zhuang Autonomous Region, P.R.CHINA. The clinicopathological parameters of the patients were recorded. All patients signed informed consent forms. This study complied with all the relevant national regulations and institutional policies and was approved by the ethics committee of The First Affiliated Hospital of Guangxi Medical University. Four tissue microarrays (ie,LVC1505, LVC1531, LVC1601, and LVC1602) were prepared, and IHC staining was performed using the MAOA antibody. All operations complied with the manufacturer's requirements.

### 2.7 Exploration of the effect of NC on *MAOA* expression

Our team has been studying the biological function NC has on HCC for a period. Previous in vitro and in vivo studies have verified that TCM NC exhibits a strong anti‐HCC capability by mediating complicated mechanisms, namely TF‐miRNA‐mRNA axis, circRNA‐miRNA‐mRNA network, andJAK1/STAT3 cascade signaling inhibition.^34^, ^35^, ^36^, ^37^, ^38^ Given the role that *MAOA* plays in HCC, we supposed that NC could exert its anti‐tumor effect partially by influencing the expression of *MAOA*. To validate our hypothesis, an in vivo experiment was conducted to study the effect of TCM NC on *MAOA* expression in a nude mouse HCC model. A total of 48 healthy nude mice were inoculated with SMMC‐7721 HCC cells (1 × 10^7^ cells/L) subcutaneously. The nude mice were divided randomly into three groups (ie NC‐treated group with intraperitoneal injection of NC [7 mg·kg^−1^], negative control group with intraperitoneal injection of saline, and the blank control group) when the tumor diameter was 15‐30 mm. After administration for 15 days, the nude mice were anesthetized and the tumor tissues were excised. Subsequently, we used next‐generation sequencing methods to determine the mRNA expression profiles in HCC tissues dissected from NC‐treated and control nude mice. The DEGs were identified using the Limma Voom package in R software v3.6.1; the criteria used were described previously. The research related to animals use complied with all the relevant national regulations and institutional policies for the care and use of animals (Ethics number: 2018‐KY‐NSFC‐102). All operations strictly complied with the manufacturer's requirements.

## 3 RESULTS

### 3.1 Included datasets and quality assessment

As shown in the flow chart (Figure S1), we retrieved datasets from the GEO, Array Express, SRA, Oncomine, TCGA, and GTEx databases. A total of 109 datasets were obtained after 48 duplicates were removed. Among these datasets, 26 were excluded, the data of which were not available. After verifying the matrices, seven datasets were removed without *MAOA* expression data. Eventually, 76 datasets were included for the analysis. To solidify the reliability of our study, the data were verified by LJD, GL, and YX independently, and a consensus was reached.

### 3.2 Dataset information

Table S1 shows the basic information of the 76 included datasets and one in‐house tissue microarray, covering 4348 HCC patients and 3624 non‐cancerous controls from 2007 to 2020. The datasets came from various ethnicities: 42 Asian datasets and 34 non‐Asian datasets. The expression levels of *MAOA* in the HCC patients and control groups were analyzed either by genechip technology (N = 58) or the RNA sequencing method (N = 18). The expression levels of *MAOA* in the HCC patients and non‐cancer groups were compared using the mean (M) expression levels and standard deviation (SD). All the specimens were hepatic tissues collected from HCC patients and non‐cancer individuals.

### 3.3 Decreased expression level of *MAOA mRNA* in HCC patients

**FIGURE 1:**
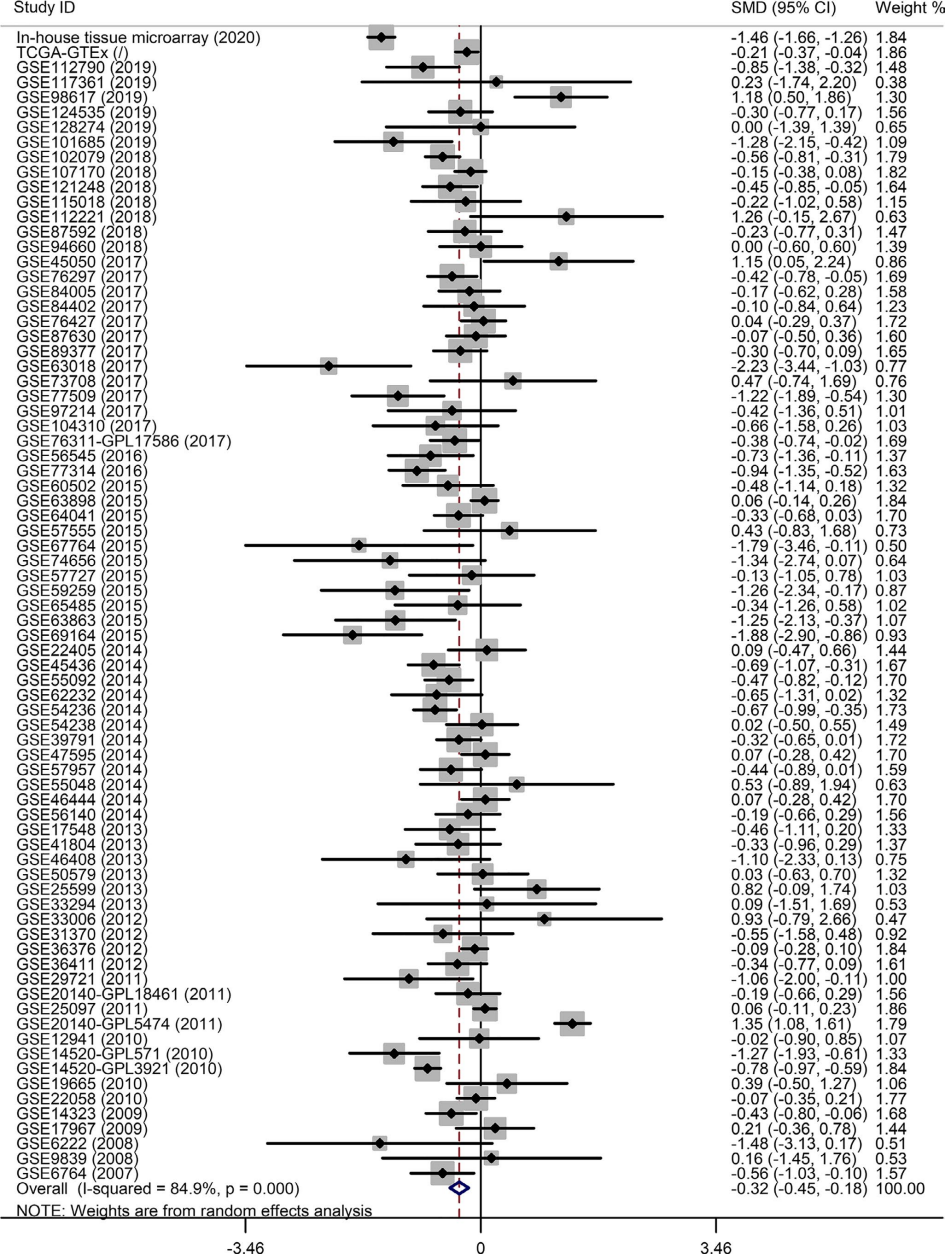
A forest plot based on the included datasets and in‐house tissue microarray. Decreased *MAOA*mRNA expression levels were found in hepatocellular carcinoma patients compared to non‐cancerous groups

The expression statuses of *MAOA* in various cancers are shown in Figure S2A. Among the RNA sequencing datasets, seven datasets showed a significant downregulation trend of *MAOA* in the HCC patients compared with the noncancer groups: GSE63018, GSE56545, GSE63863, GSE77509, GSE77314, TCGA‐GTEx, and GSE69164 (all with *P*‐values <.05; Figure S2B‐Q). As for the genechip datasets, the scatter plots in Figure S3A‐P show significant lower expression levels of *MAOA* in the HCC patients compared with the non‐cancer groups in GSE6764, GSE14323, GSE14520‐GPL571, GSE14520‐GPL3921, GSE29721, GSE45436, GSE55092, GSE76297, GSE102079, GSE112790, GSE121248, GSE54236, GSE67764, and GSE59259 (all with *P*‐values <.05). Subsequently, the heterogeneity test of the included datasets was conducted, and a result of *I* ^2^ = 84.9% (*P*‐value < .0001) indicated a significant heterogeneity. Thus, a random effects analysis was employed to compute the SMD. The outcome exhibited in the *MAOA* expression levels in patients with HCC was significantly lower than that in the non‐cancer groups (Figure 1, SMD = −0.32, 95% CI: −0.45 to −0.18). Moreover, the outcome of the sensitive analysis showed no significant difference among the included studies (Figure S4A). The Begg's funnel plot indicated no publication bias, with a *P*‐value = .657 (Figure S4B). Two subgroup analyses were performed to assess the expression status of *MAOA* from two perspectives (Figures S5 and S6).The results revealed that neither ethnicity nor the methods used to determine the *MAOA* mRNA expression levels of HCC patients was the source of heterogeneity in the present study, as *MAOA* was downregulated in all subgroups. To validate the *MAOA* expression status in HCC, we also downloaded the RNA sequencing data from the Broad Institute Cancer Cell Line Encyclopedia (CCLE). Surprisingly, we found that *MAOA* was not expressed in the cell lines of C3A_LIVER, ALEXANDERCELLS_LIVER, and HLE_LIVER. Therefore, the evidence confirmed the downregulated *MAOA* level in HCC.

### 3.4 Promising diagnostic and prognostic value of downregulated *MAOA* in HCC

**FIGURE 2:**
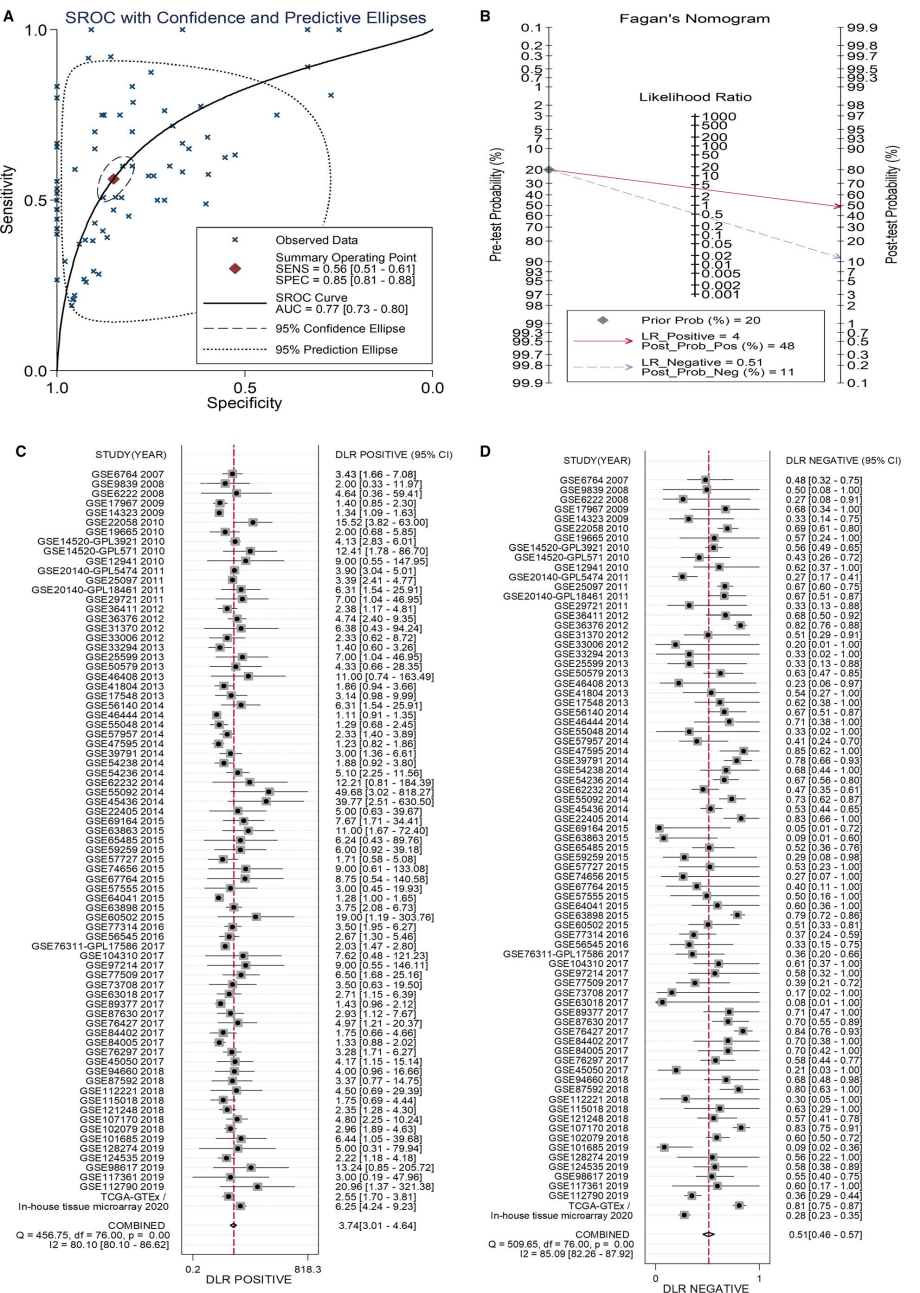
Hepatocellular carcinoma (HCC) patient diagnostic trial by using *MAOA* as a marker. A, The summary receiver operating characteristic (sROC) curve exhibited a marvelous discriminatory capacity of *MAOA* in HCC and non‐HCC patients. B, Fagan's nomogram, (C) diagnostic likelihood ratio negative (DLR N), and (D) diagnostic likelihood ratio positive (DLR N) also addressed the diagnostic value of *MAOA* for HCC patients

**FIGURE 3:**
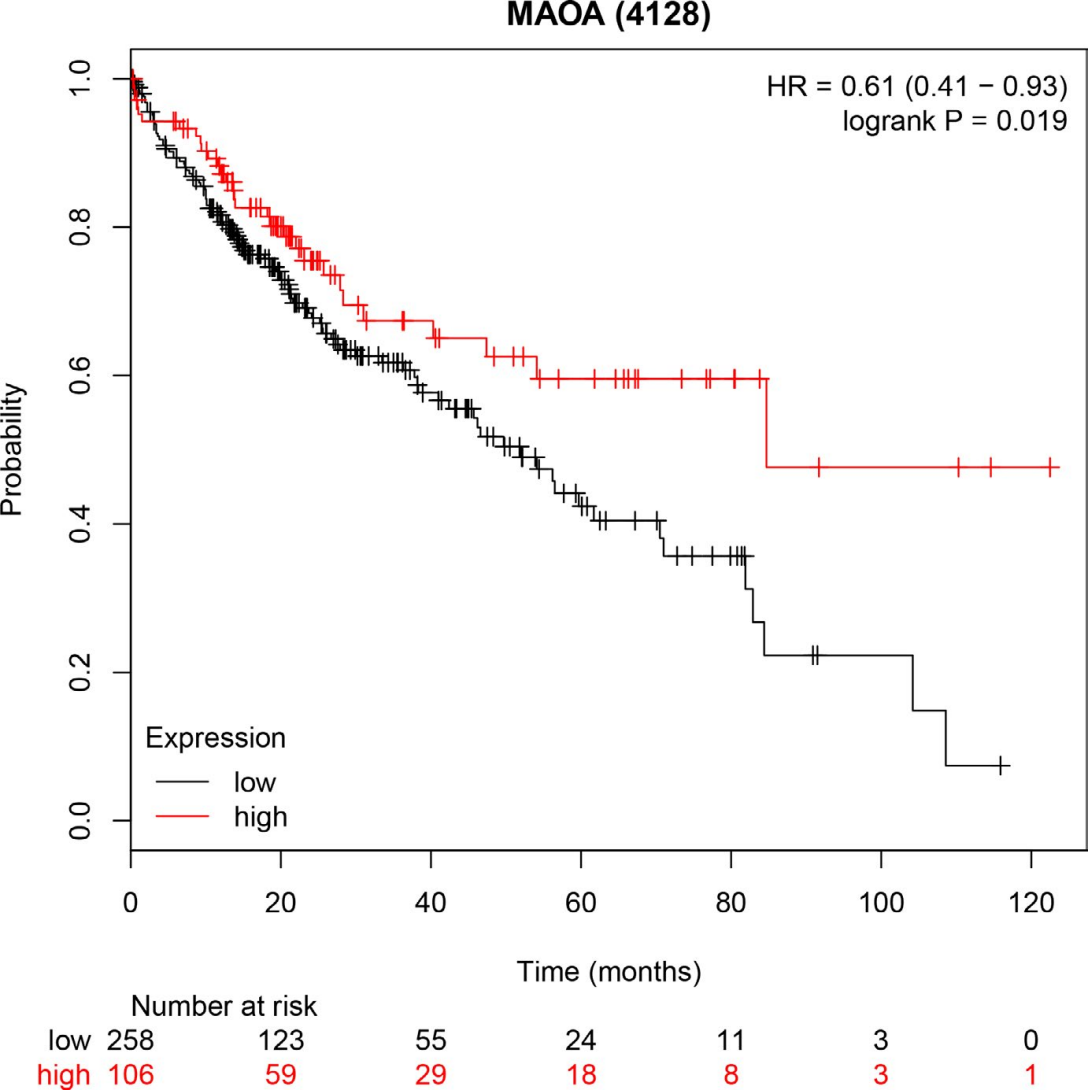
A Kaplan‐Meier curve of the hepatocellular carcinoma patients. The result indicated that the low *MAOA* group possessed worse survival outcome

The ROC curves of the following six RNA sequencing datasets indicated the discriminatory capacity of *MAOA* in the HCC patients and non‐HCC groups: GSE63018, GSE56545, GSE63863, GSE77509, GSE77314, and GSE69164 (all with AUC >0.70 and *P*‐values <.05; Figure S7A‐P). Regarding the gene chip dataset, the remarkable distinguishing ability of *MAOA* in the HCC patients and non‐cancer individuals was exhibited in GSE14520‐GPL571, GSE14520‐GPL3921, GSE29721, GSE45436, GSE112790, and GSE59259 (all with AUC >0.70 and *P*‐values <.05; Figure S8A‐I). The AUC of the sROC curve, sensitivity, and specificity were 0.77 (95% CI: 0.73‐0.80), 0.56 (95% CI: 0.51‐0.61), and 0.85 (95% CI: 0.81‐0.88), respectively (Figure 2A). In the Fagan plot, with a pre‐test probability of 20%, the post‐test probability of HCC using *MAOA* for a positive test result was 48%, and the probability of a negative test result was 11%, suggesting that *MAOA* could serve as a remarkable marker for HCC screening (Figure 2B). The diagnostic likelihood ratio positive (DLR P), diagnostic likelihood ratio negative (DLR N), diagnostic score, and diagnostic odd ratio were 3.74 (95% CI: 3.01‐4.64), 0.51 (95% CI: 0.46‐0.57), 1.98 (95% CI: 1.73‐2.23), and 7.26 (95% CI: 5.64‐9.34) respectively (Figure 2C and D).The aforementioned outcomes indicated that *MAOA* had an excellent discriminatory ability for HCC. Furthermore, the downregulated expression level of *MAOA* was relevant to age (≥ 60), race (Asian), tumor metastasis status (M0), neoplasm histologic grade (G1>G2>G3>G4), and AFP concentration (>20 ng/mL) (all with *P*‐values <0.05; Table S2; Figure S9A‐E). Interestingly, the Kaplan‐Meier curve indicated that the high *MAOA* mRNA expression group had a better survival outcome, with a hazard ratio of 0.61 (95% CI: 0.41‐0.93) (Logrank *P* = .019; Figure 3), indicating that the downregulated *MAOA*mRNA correlated with a poor prognosis of HCC patients.

### 3.5 Genetic alterations and mutation types of *MAOA* in HCC

**FIGURE 4:**
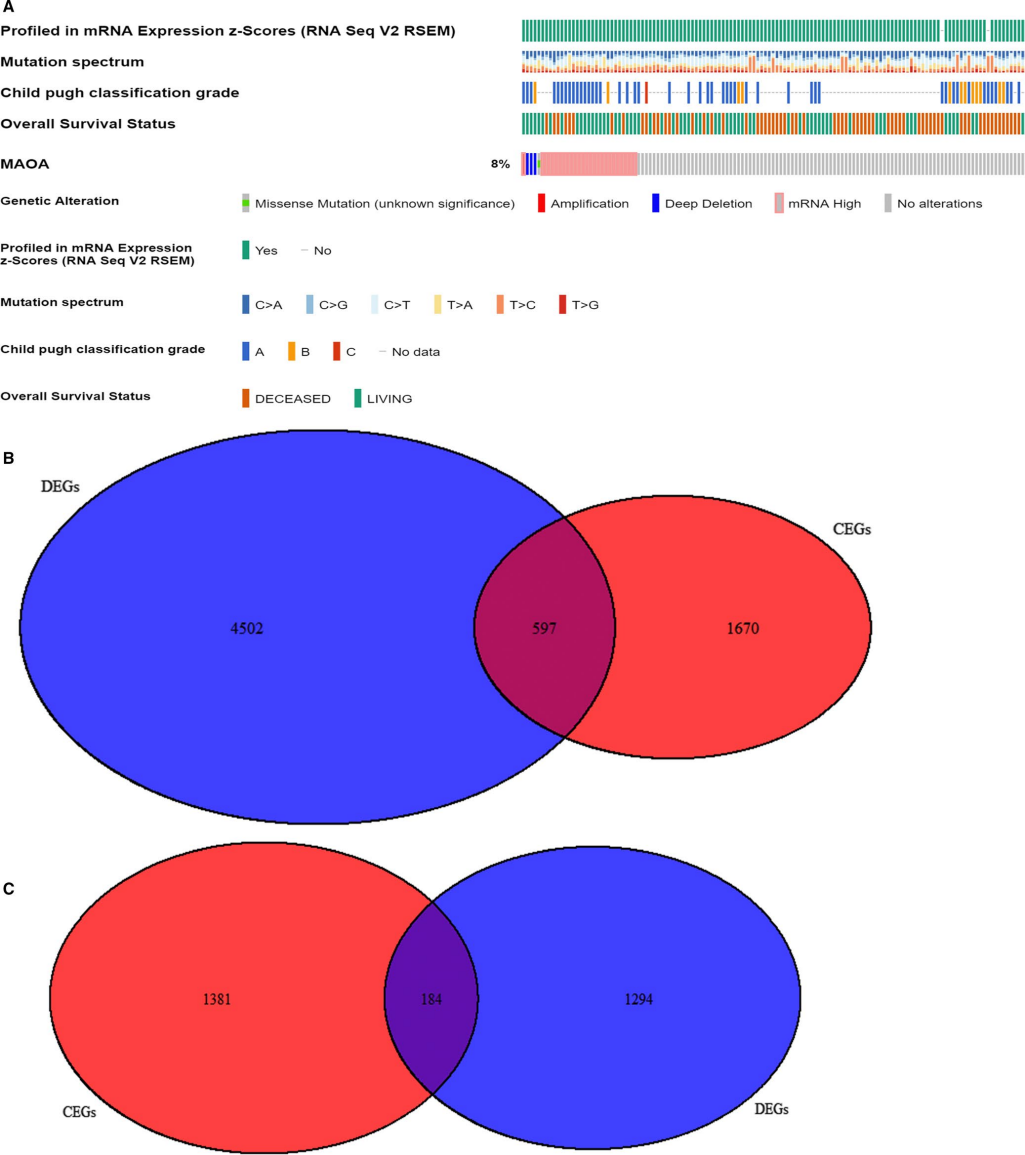
*MAOA* genetic alteration status based on cBioPortal. A, *MAOA* is altered in 30 (8%) of the queried hepatocellular patients. B, Venn diagram for the intersection genes of 5099 *MAOA* positively related co‐expressed genes (CEGs) and 2267 downregulated differentially expressed genes (DEGs). C, Venn diagram for the intersection genes of 1565 *MAOA*‐negatively‐related CEGs and 1478 up‐regulated DEGs

*MAOA* was altered in 30 (8%) of the 440 patients in the study on liver hepatocellular carcinoma (TCGA, Provisional) (Figure 4A). Notably, not all genetic alteration information of the patients was available. The genetic alterations of *MAOA* in HCC were focused on missense mutation, amplification, deep deletion, and the mRNA overexpression level. Further study on the mutation type of *MAOA* in HCC showed that missense was the predominant mutation type (Table S3).

### 3.6 Identification of CEGs of *MAOA* and DEGs

The Pearson's correlation coefficient of *MAOA* and other genes in each dataset was computed. Two correlation heatmaps were plotted based on the calculated Pearson's correlation coefficients of GSE6222 and GSE19665 (Figure S10A and B). Subsequently, the co‐expressed genes meeting the standards of the absolute value of Pearson's correlation coefficient >0.3 and a *P*‐value <.05 were obtained for further analysis. A total of 5099 *MAOA* positively related CEGs and 1565 *MAOA* negatively related CEGs were identified, all of which appeared in no fewer than nine datasets. Herein, *PCOLCE* and *TBC1D8* were detected to be the most significant CEGs positively related to *MAOA*, and *PKM* was detected to be the most significant CEGs positively related to *MAOA* (Figure S11A and B). In addition, 2267 downregulated genes and 1478 upregulated genes appearing in no fewer than eight datasets were identified. In total, 597 intersection genes of the *MAOA* positively related CEGs and downregulated DEGs and 184 intersection genes of the *MAOA* negatively related CEGs and upregulated DEGs were obtained (Figure 4B and C).

### 3.7 GO, DO, and KEGG enrichment analyses and PPI network construction

**FIGURE 5:**
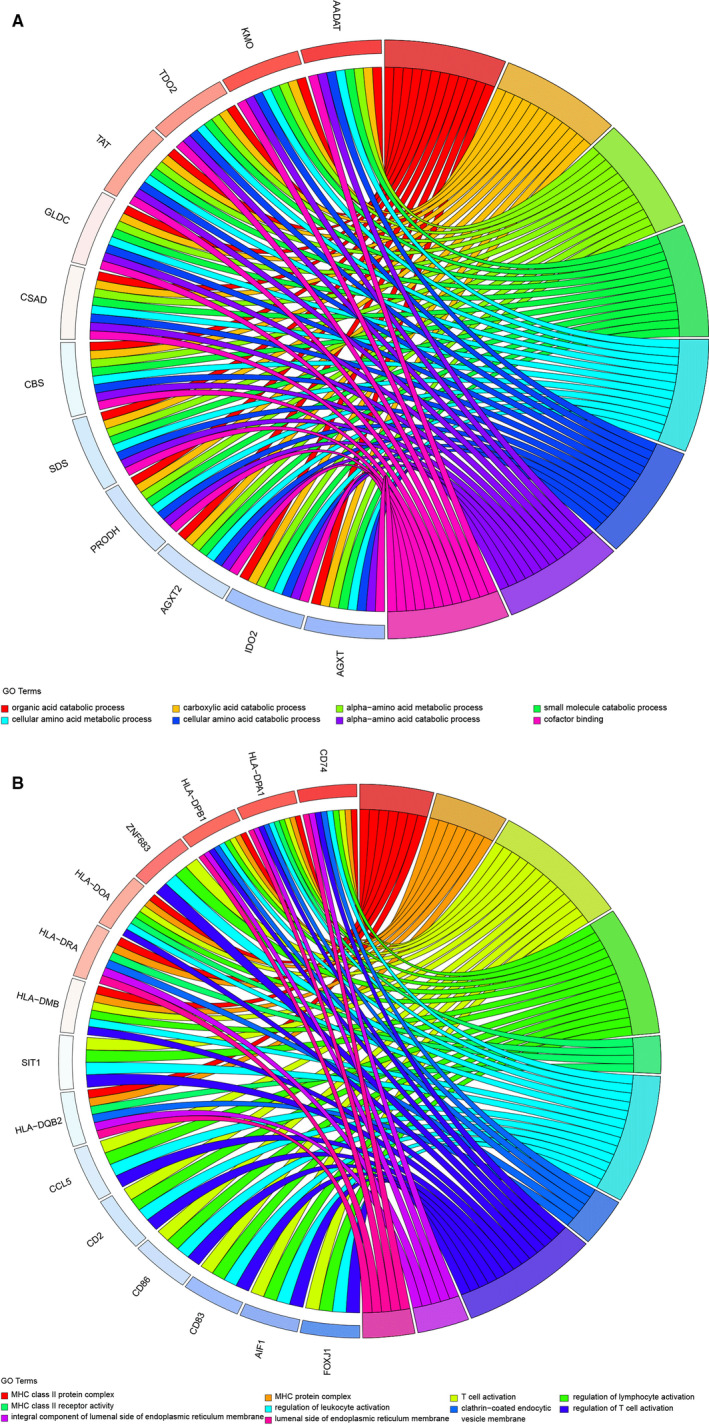
GO analysis based on the intersection genes. A, Chord plot of the 597 intersection genes from *MAOA* positively related co‐expressed genes (CEGs) and downregulated differentially expressed genes (DEGs). B, Chord plot of the 184 intersection genes of *MAOA* negatively related CEGs and upregulated DEGs. GO, Gene Ontology

**FIGURE 6:**
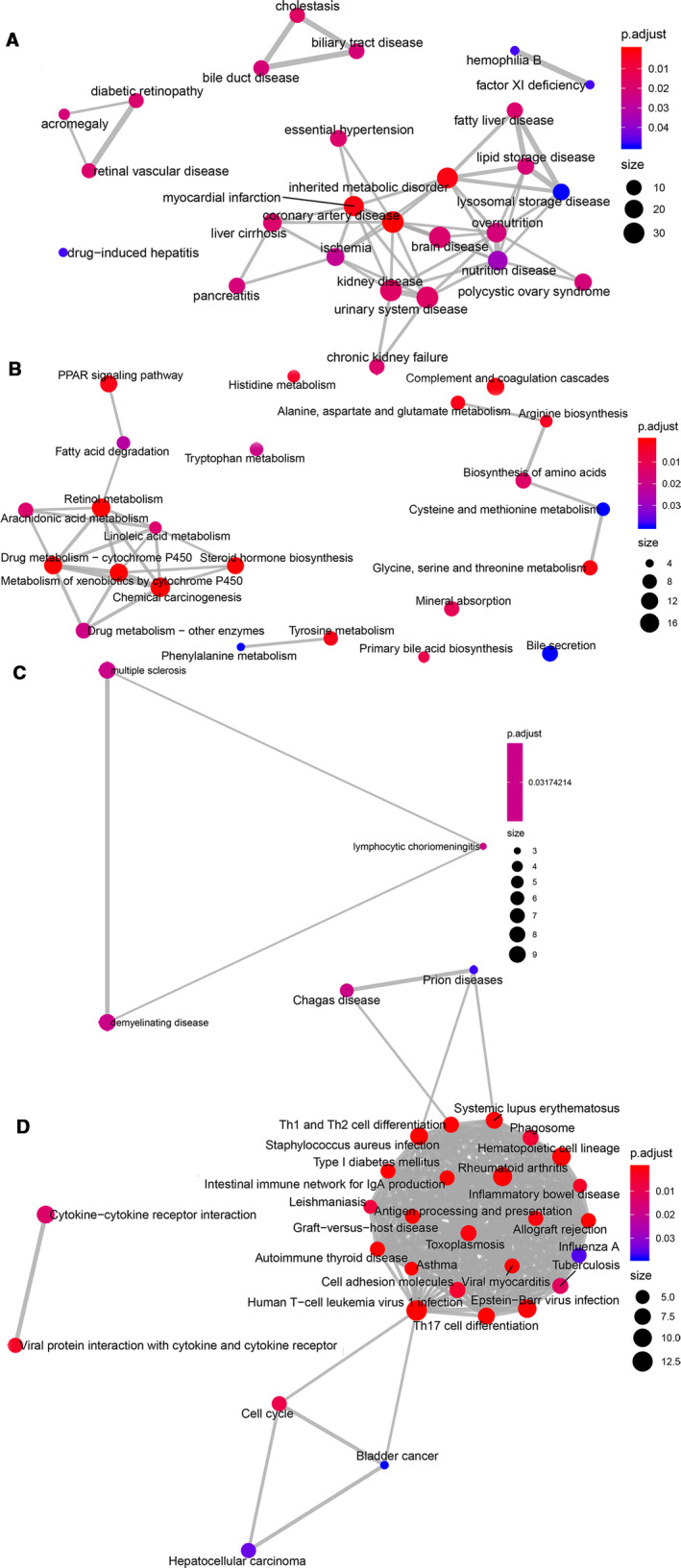
DO enrichment analysis and KEGG pathway. A, DO and (B) KEGG emap plot of the 597 intersection genes from *MAOA* positively related co‐expressed genes (CEGs) and downregulated differentially expressed genes (DEGs). (C) DO and (D) KEGG emap plot of the 184 intersection genes of *MAOA* negatively related CEGs and upregulated DEGs. DO, Disease Ontology. KEGG, the Kyoto Encyclopedia of Genes and Genomes

According to the 597 intersection genes of the *MAOA* positively related CEGs and downregulated DEGs, GO analysis showed the importance of the organic acid catabolic process, blood microparticle, and monooxygenase activity in terms of biological process (BP), cellular component (CC), and molecular function (MF) respectively (Figure 5A; Table S4). For the 184 intersection genes of the *MAOA* negatively related CEGs and upregulated DEGs, T cell activation, MHC class II protein complex, and MHC class II receptor activity were emphasized in terms of BP, CC, and MF respectively (Figure 5B; Table S5). Based on the 597 intersection genes, DO analysis and KEGG enrichment analysis indicated that coronary artery disease (Figure 6A) was the predominantly associated disease and that retinol metabolism and chemical carcinogenesis were the top two clustered pathways (Figure 6B). We wanted to focus on the chemical carcinogenesis pathway. Thus, we analyzed the specific process of the chemical carcinogenesis pathway (Figure S12) and found that *MAOA* could participate in the pathogenesis and progression of HCC and other cancers through multiple genes, namely CYP1A1, CYP1A2, CYP2A, CYP2E1, CYP3A4, GST, HSD11B1, NAT, NAT2, and UGT. Among these genes, CYP3A4 played an important role in the carcinogenesis process of HCC by promoting the transformation from aflatoxin B1 to AFB1‐exo‐8,9‐epoxide. The 184 intersection genes of the *MAOA* negatively related CEGs and upregulated DEGs were significantly related to lymphocytic choriomeningitis (Figure 6C) and were primarily enriched in the KEGG pathway of rheumatoid arthritis (Figure 6D). Moreover, PPI networks were constructed based on the genes in the chemical carcinogenesis pathway to show the interplay of proteins (Figure S13). CYP2E1 was selected as the hub gene in the network. We also explored the prognostic value of CYP2E1, and, consistent with *MAOA*, the results indicating low CYP2E1 correlated with the reduced overall survival in HCC patients (Figure S14A and B).

### 3.8 Protein expression level and clinical significance of MAOA in HCC

**FIGURE 7:**
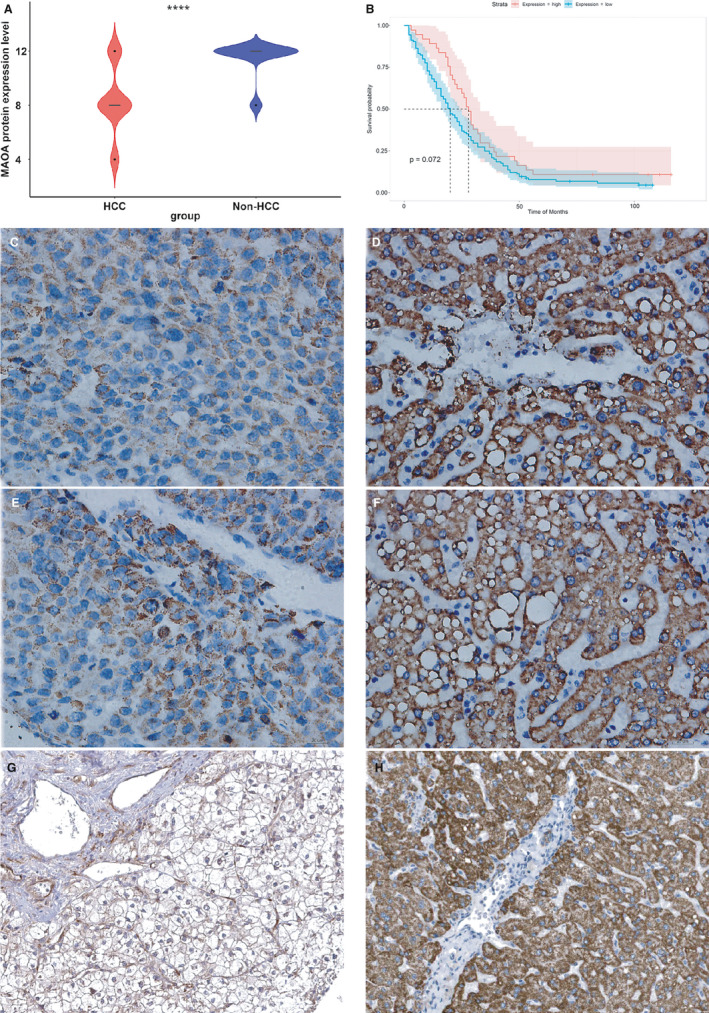
Expression level and clinical significance of MAOA protein in hepatocellular carcinoma (HCC). A, MAOA protein expression was significantly decreased in HCC compared to non‐HCC tissues. B, A Kaplan‐Meier curve of the hepatocellular carcinoma patients based on the MAOA protein expression level. However, no significant difference was identified between low and high MAOA protein expression group in HCC patients. HCC (C, E, and G)and non‐HCC (D, F, and H) immunohistochemical (IHC) staining images under a microscope (x400) signified lower MAOA protein expression in HCC compared to non‐HCC tissues. G and H were provided by HumanProteinAtlas (antibody: CAB009437)

The results of the tissue microarrays and IHC staining indicated that the MAOA protein expression level was significantly decreased in HCC compared with non‐HCC specimens (Figure 7A), consistent with the expression status of *MAOA* from the mRNA level. A Kaplan‐Meier curve of the hepatocellular carcinoma patients based on the MAOA protein expression level was drawn, and no significant difference was identified between the low and high MAOA protein expression groups in HCC patients (Figure 7B). The HCC (Figure 7C, E, and G) and non‐HCC (Figure 7D, F, and H) immunohistochemical IHC staining images under a microscope (×400) indicated a lower MAOA protein expression in HCC compared with non‐HCC tissues, as confirmed by the IHC staining results from Human Protein Atlas

(Figure 7G and H). More importantly, we found that a lower MAOA protein expression was significantly correlated with the advanced TNM stage of HCC patients (Table S6).

### 3.9 Effect of NC on the *MAOA* expression in the nude mouse HCC model

The tumor volumes of the NC‐treated nude mice were significantly reduced when compared with control nude mice (*P*‐value <.05).^39^ A total of two NC‐treated and three control HCC tissues were eligible for expression analysis. The mRNA expression profiles in HCC tissues dissected from NC‐treated and control nude mice were determined by next‐generation sequencing. The DEGs were identified, and a volcano plot was drawn (Figure S15). As expected, the *MAOA* expression level was significantly elevated in the NC‐treated group compared with the control group.

## 4 DISCUSSION

In this study, we validated the downregulation of *MAOA* and evaluated its clinical values. We revealed the impact NC has on *MAOA* for the first time and explored its potential mechanism.

When compared with the previous studies of *MAOA* concerning HCC, our research appraised the clinical value of *MAOA* in a more comprehensive manner. As a multiple‐centered study, we have integrated in‐house tissue chip with large‐scale of microarrays and sequencing data sets of HCC tissues. We confirmed that *MAOA* expression exhibited a downregulated trend after analyzing 4033 HCC and 3432 controls, and the decreased level of *MAOA* in HCC displayed no significant association with ethnicity or expression profile methods (namely gene chip and high throughout sequencing).

According to the study of Du et al, *MAOA* mediates a signaling pathway contributing to reactive oxygen species generation, through which cancer‐associated fibroblasts induce the epithelial‐mesenchymal transition, metastasis, and infiltration of prostate cancer.^40^ Liao etal demonstrated that *MAOA* promoted prostate tumor cell proliferation by mediating AKT phosphorylation and that *MAOA* absence hampered the progression of prostate cancer.^41^ In the present study, we found that decreased *MAOA* in HCC correlated with poor survival outcome. Therefore, MAOA could be a potential prognosis predictor of HCC patients.

We identified the CEGs and DEGs of *MAOA* in HCC for further analysis of the mechanisms. *PCOLCE* and *TBC1D8* were selected as the most significant CEGs positively related to *MAOA*, and *PKM* was detected as the most significant CEGs negatively related to *MAOA*. According to Li et al, *PCOLCE* was significantly downregulated in liver cancer and was positively correlated with *DAPK1*, which is a proven prognostic factor of liver cancer.^42^ Although research on *TBC1D8* in HCC is lacking, it has been found to be an independent prognosis marker in ovarian cancer. It was reported that circMAT2Bcould accelerate the progression of HCC by miR‐338‐3p/PKM2 axis.^43^ A GO enrichment analysis based on the 597 intersection genes of the *MAOA* positively related CEGs and downregulated DEGs emphasized the importance of the organic acid catabolic process, blood microparticle, and cofactor binding in the GO terms of BP, CC, and MF respectively. These genes were related to coronary artery disease and were prominently clustered in the chemical carcinogenesis pathway. We found downregulated DEGs that positively correlated with *MAOA*, especially *CYP1A1*, *CYP1A2*, *CYP2A*, *CYP2E1*, *CYP3A4*, *GST*, *HSD11B1*, *NAT*, *NAT2*, and *UGT*, were significantly aggregated in the chemical carcinogenesis pathway. Interestingly, *CYP1A1*, *CYP1A2*, *CYP2A*, *CYP2E1*, and *CYP3A4* all belong to genes that encode the essential enzymes of the cytochrome P450 system and participate in the metabolism and biological transformation of chemicals.^44^, ^45^, ^46^, ^47^ Among these genes, *CYP3A4*, which is highlighted in the KEGG pathway, played an important role in the carcinogenesis process of HCC by promoting the transformation from aflatoxin B1 to AFB1‐exo‐8,9‐epoxide, consistent with Bonomo et al.^48^ There is a consensus that AFB1,^49^ the most powerful carcinogen to date, works as a trigger in the carcinogenesis of the liver. In this study, co‐expression analysis revealed the positive relation between *MAOA* and *CYP3A4*. Recently, it has been confirmed that MAOA and CYP3A4 were two major enzymes that participated in the formation of metabolite A and D in hepatocytes.^50^ Therefore, it can be inferred that *MAOA* may synergistically participate in the toxic transformation of AFB1 through its positively related CEGs, especially *CYP3A4*, thus causing pathogenesis and progression in HCC patients. Our findings enrich the understanding of pathogenesis and progression of HCC. More studies are needed to further confirm the interaction between *MAOA* and *CYP3A4* in the future.

Regarding the 184 intersection genes of the *MAOA* negatively related CEGs and upregulated DEGs, T cell activation, MHC class II protein complex, and MHC class II receptor activity were important in the GO terms of BP, CC, and MF, respectively. These genes were significantly associated with lymphocytic choriomeningitis in DO analysis and were enriched in the KEGG pathway of rheumatoid arthritis. To the best of our knowledge, cytotoxic T lymphocyte (CTL) is considered the most pivotal effector cell in anti‐tumor immunity,^51^ and it is capable of specifically killing tumor cells through the Fas‐FasL and TNF‐TNFR pathway and the perforin/granzyme pathway. The activation of CTL relies on two signals: tumor antigen presented by the antigen presentation cell (APC) and co‐stimulatory molecules, especially CD28, located on the surface of the T cell.^52^ MHC class II protein complex, a molecule distributed to APC and activated T cell, plays an important role in the indirect activation of CD8^+^ T cells by presenting the tumor antigen to CD4^+^ T cells. With the help of CD4^+^ T cells, CD8^+^ T cells successfully differentiate into CTL and exert its anti‐tumor effects. Contrary to the carcinogenesis effect of *MAOA* positively related CEGs, the *MAOA* negatively related CEGs exhibit an anti‐tumor biological function by promoting T cell activation while participating in coding the MHC class II protein complex. Therefore, we suppose that the imbalance between the biological functions of *MAOA* positively related CEGs and those of *MAOA* negatively related CEGs may partially account for the onset of HCC.

Interestingly, we found that NC could exert its partial anti‐tumor effect by influencing the expression of *MAOA*. As demonstrated previously, a decreased *MAOA* was detected in HCC patients and correlated with poor prognosis. Our previous studies have confirmed the anti‐HCC effect of NC.^34^, ^35^, ^37^ In this study, in vivo experiment showed that the tumor volumes were reduced and the *MAOA* expression level was significantly elevated in the NC‐treated group compared with the control group. Taken together, NC increases MAOA expression and plays a protective role in HCC nude mouse model. To date, some mechanisms of the suppressor role of NC in HCC have been proved. Based on in vitro and in vivo studies, TF‐miRNA‐mRNA axis and circRNA‐miRNA‐mRNA network were established to provide clues on the role of NC in HCC.^34^, ^35^ Another in vitro and in vivo experiment confirmed that the therapeutic effect of NC on HCC correlated with the upregulation of p53 and Bcl‐2.^36^ According to an in vivo study of Jun Liao etal, the tumor suppression effect of NC was also attributed to the JAK1/STAT3 cascade signaling inhibition.^38^ In light of the present study, it is reasonable to suppose that NC may attenuate tumor cell growth and other malignant behaviors in HCC by increasing the *MAOA* expression.

The unique advantages of the present study are as follows. First, the clinical value of *MAOA* was comprehensively estimated by integrating in‐house tissue microarray, genechip, and RNA sequencing data. We not only discovered the moderate capability of *MAOA* in distinguishing HCC from non‐HCC patients but also showed the relevance between a downregulated *MAOA* and the poor prognosis of HCC patients. Second, a decreased *MAOA* expression was verified by immunohistochemical staining, and a downregulated MAOA protein expression was correlated with an advanced TNM stage in HCC patients. Third, we found that TCM NC showed a potential in anti‐HCC by upregulating *MAOA*.

Nevertheless, this study has its limitations. First, the included datasets had high heterogeneity, and the influence analysis indicated no significant difference. The reasons for this could be due to the different methods and platforms used in the datasets and the patients coming from various countries, such as Singapore, China, the United States, Switzerland, Japan, Turkey, Japan, Canada, South Korea, Spain, Germany, and Italy. To cope with the high heterogeneity, a randomized effect model was employed in our analysis. Second, *MAOA* had a sensitivity of 0.56 (95% CI: 0.51‐0.61), which is slightly lower than that of AFP (63.3%). Therefore, *MAOA* is more effective in confirming diagnosis than in screening in HCC. Third, our result was based on clinical data and in vivo mouse model. Therefore, large‐scale clinical experiments are needed to further confirm the diagnostic and prognostic significance of *MAOA* in HCC in the future, and the potential therapeutic effect of NC by targeting *MAOA* should be further validated.

In conclusion, a decreased *MAOA* level is not only correlated with aggressive behaviors in males but also serves as a promising biomarker for the diagnosis and prognosis of HCC patients. Moreover, *MAOA* may play a role in AFB1 toxic transformation through its synergistic action with co‐expressed genes, especially CYP3A4. *MAOA* also serves as a potential therapy target of NC in HCC patients.

## CONFLICT OF INTEREST

The authors declare no conflict of interest.

## AUTHOR CONTRIBUTIONS

Design of project study, experiment operation directing, statistics process directing, and modification of paper: Yu‐Yan Pang, Yi‐Wu Dang, Ze‐Feng Lai, Li‐Min Liu, Jie Yang, Hua‐Yu Wu, Rong‐Quan He, Zhi‐Guang Huang, Li‐Hua Yang, Lin Shi, Wei‐Jia Mo, Deng Tang, Hui‐Ping Lu, and Gang Chen; experiment operation of tissue microarray immunohistochemistry, data collection, data analysis, and manuscript drafting: Jian‐Di Li, Li Gao, Xia Yang, and Dan‐Dan Xiong; final approval of manuscript: all the authors.

## Supporting information

Figure S1
Figure S2
Figure S3
Figure S4
Figure S5
Figure S6
Figure S7
Figure S8
Figure S9
Figure S10
Figure S11
Figure S12
Figure S13
Figure S14
Figure S15
Table S1‐S6

## Data Availability

The original data are available from the corresponding author upon request.
