# Supplementary material for: The clinical value and potential molecular mechanism of the downregulation of MAOA in hepatocellular carcinoma tissues
Source: Cancer Med. 2020 Sep 15;9(21):8004–19. doi: 10.1002/cam4.3434 (PMC7643659; doi:10.1002/cam4.3434)
Supplement: Supplementary file 1 — Figure S1 Figure S2 Figure S3 Figure S4 Figure S5 Figure S6 Figure S7 Figure S8 Figure S9 Figure S10 Figure S11 Figure S12 Figure S13 Figure S14 Figure S15 Table S1‐S6 [file CAM4-9-8004-s001.pdf]

Supplementary Tables

Supplementary Table 1. Basic information of the included external datasets. HCC, hepatocellular carcinoma; N, number; M, mean; SD, standard deviation; TP, true positive; FP, false positive; FN, false negative; TN, true negative.

**Accession**	**Author**	**Year**	**Platform**	**Country**	**HCC**			**Non-cancer**			**TP**	**FP**	**FN**	**TN**
					**N1**	**M1**	**SD1**	**N2**	**M2**	**SD2**				
**TCGA-GTEx**	/	/	/	/	371	12.50	1.19	225	12.71	0.64	105	25	266	200
**GSE112790**	Kaoru Mogushi, et al.	2019	GPL570	Japan	183	9.13	0.90	15	9.87	0.25	120	0	63	15
**GSE117361**	Yong Jiang, et al.	2019	GPL6480	China	2	9.11	1.82	2	8.78	0.94	1	0	1	2
**GSE98617**	Sara Torrecilla, et al.	2019	GPL14951	Spain	36	14.06	0.63	13	12.96	1.50	17	0	19	13
**GSE124535**	Ying Jiang, et al.	2019	GPL20795	China	35	5.32	0.81	35	5.51	0.40	20	9	15	26
**GSE128274**	Ge Shan, et al.	2019	GPL18573	China	4	4.74	0.61	4	4.74	0.19	2	0	2	4
**GSE101685**	Yun-Shien Lee, et al.	2019	GPL570	China	24	8.17	0.92	8	9.24	0.45	23	1	2	6
**GSE102079**	Kaoru Mogushi, et al.	2018	GPL570	Japan	152	9.02	0.94	105	9.46	0.48	77	18	75	87
**GSE107170**	Giacomo Diaz, et al.	2018	GPL570	Italy	124	9.51	0.86	183	9.61	0.48	26	8	98	175
**GSE121248**	Kam Hui, et al.	2018	GPL570	Singapore	70	9.46	0.90	37	9.80	0.34	40	9	30	28
**GSE115018**	Guang-Qiang Ye, et al.	2018	GPL20115	China	12	1.72	1.23	12	1.93	0.56	7	4	5	8
**GSE112221**	Keith D Robertson, et al.	2018	GPL16791	USA	4	4.41	0.67	6	3.62	0.60	3	1	1	5
**GSE87592**	Shi-Long Zhong, et al.	2018	GPL11154	China	27	5.45	0.34	26	5.53	0.35	7	2	20	24
**GSE94660**	Seungyeul Yoo, et al.	2018	GPL16791	USA	21	4.20	0.88	21	4.20	0.27	8	2	13	19
**GSE45050**	Moses Morakortoi Darpolor, et al.	2017	GPL6244	USA	6	11.00	0.54	10	10.51	0.35	5	2	1	8
**GSE76297**	Xin-Wei Wang, et al.	2017	GPL17586	USA	61	7.96	0.71	58	8.19	0.30	31	9	30	49
**GSE84005**	Jin Song, et al.	2017	GPL5175	China	38	10.26	0.96	38	10.38	0.33	24	18	14	20
**GSE84402**	Zhuo-An Cheng, et al.	2017	GPL570	China	14	8.10	1.09	14	8.19	0.74	7	4	7	10
**GSE76427**	Surya Pavan Yenamandra, et al.	2017	GPL10558	Singapore	115	12.11	0.79	52	12.08	0.57	22	2	93	50
**GSE87630**	Hyun Goo Woo, et al.	2017	GPL6947	South Korea	64	11.89	0.68	30	11.93	0.21	25	4	39	26
**GSE89377**	Jungwoo Eun, et al.	2017	GPL6947	South Korea	40	11.50	0.87	67	11.69	0.43	23	27	17	40
**GSE63018**	Bic Mskcc, et al.	2017	GPL16791	USA	9	11.55	0.67	9	12.98	0.61	9	3	0	6
**GSE73708**	Jian Chen, et al.	2017	GPL11154	USA	8	3.49	0.94	4	3.08	0.66	7	1	1	3
**GSE77509**	Jin Gu, et al.	2017	GPL16791	China	20	13.20	0.79	20	13.97	0.42	13	2	7	18

Accession	Author	Year	Platform	Country	HCC			Non-cancer			TP	FP	FN	TN
					N1	M1	SD1	N2	M2	SD2				
**GSE97214**	Yi Huang, et al.	2017	GPL11154	China	9	4.80	1.14	9	5.17	0.48	4	0	5	9
**GSE104310**	Jing-Ping Yun, et al.	2017	GPL16791	China	12	4.46	0.94	8	4.99	0.50	5	0	7	8
**GSE76311-GPL17586**	Xin-Wei Wang, et al.	2017	GPL17586	USA	62	7.98	0.71	59	8.19	0.30	31	31	9	50
**GSE56545**	Dong Shen, et al.	2016	GPL15433	USA	21	10.37	1.13	21	11.05	0.66	16	6	5	15
**GSE77314**	Gang Liu, et al.	2016	GPL9052	China	50	4.48	1.12	50	5.34	0.66	35	10	15	40
**GSE60502**	K.J. Kao, et al.	2015	GPL96	China	18	9.61	0.86	18	9.91	0.20	9	0	9	18
**GSE63898**	Augusto Villanueva, et al.	2015	GPL13667	USA	228	8.37	0.61	168	8.34	0.36	61	12	167	156
**GSE64041**	Zuzanna Makowska, et al.	2015	GPL6244	Switzerland	60	9.63	0.71	65	9.82	0.43	45	38	15	27
**GSE57555**	Y-H. Taguchi, et al.	2015	GPL16699	Japan	5	0.68	0.76	5	0.44	0.24	3	1	2	4
**GSE67764**	Bin Wu, et al.	2015	GPL17077	China	3	2.04	0.97	6	3.04	0.25	2	0	1	6
**GSE74656**	Hui-Yong Yin, et al.	2015	GPL16043	China	5	7.60	1.32	5	8.85	0.10	4	0	1	5
**GSE57727**	Manel Esteller, et al.	2015	GPL14951	Spain	57	11.28	1.01	5	11.41	0.49	39	2	18	3
**GSE59259**	Alberto Ferrarini, et al.	2015	GPL18451	Italy	8	12.33	0.80	8	13.09	0.30	6	1	2	7
**GSE65485**	Hua Dong, et al.	2015	GPL11154	China	50	4.69	1.23	5	5.09	0.26	26	0	24	5
**GSE63863**	Hong Zhang, et al.	2015	GPL11154	China	12	4.10	1.01	12	5.05	0.36	11	1	1	11
**GSE69164**	Hong Zhang, et al.	2015	GPL11154	China	11	3.90	0.76	11	5.02	0.36	11	1	0	10
**GSE22405**	Henry H Zhang, et al.	2014	GPL10553	USA	24	7.72	1.17	24	7.63	0.74	5	1	19	23
**GSE45436**	Jui-Yu Hsieh, et al.	2014	GPL570	China	93	8.04	0.98	41	8.62	0.34	44	0	49	41
**GSE55092**	Patrizia Farci, et al.	2014	GPL570	USA	49	9.46	1.00	91	9.78	0.43	13	0	36	91
**GSE62232**	Sandrine Imbeaud, et al.	2014	GPL570	France	81	8.77	0.96	10	9.36	0.21	45	0	36	10
**GSE54236**	Erica Villa, et al.	2014	GPL6480	Italy	81	11.57	0.91	80	12.05	0.44	31	6	50	74
**GSE54238**	Jie Wang, et al.	2014	GPL16955	USA	26	11.02	1.28	30	10.99	1.64	13	8	13	22
**GSE39791**	Sangbae Kim, et al.	2014	GPL10558	USA	72	10.26	1.16	72	10.56	0.63	21	7	51	65
**GSE47595**	Yujin Hoshida, et al.	2014	GPL13369	USA	88	11.15	2.54	48	10.97	2.86	43	19	45	29
**GSE57957**	Lee Guat Lay Caroline, et al.	2014	GPL10558	Singapore	39	12.07	0.70	39	12.32	0.40	28	12	11	27
**GSE55048**	Tao Cai, et al.	2014	GPL9115	China	4	5.12	0.84	4	4.77	0.42	4	3	0	1
**GSE46444**	Yujin Hoshida, et al.	2014	GPL15314	USA	88	11.15	2.54	48	10.97	2.86	71	35	17	13

Accession	Author	Year	Platform	Country	HCC			Non-cancer			TP	FP	FN	TN
					N1	M1	SD1	N2	M2	SD2				
**GSE56140**	Yujin Hoshida, et al.	2014	GPL13825	USA	35	11.30	0.71	34	11.40	0.25	13	2	22	32
**GSE17548**	Mehmet Ozturk, et al.	2013	GPL570	Turkey	17	9.07	1.07	20	9.45	0.56	8	3	9	17
**GSE41804**	Yuji Hodo, et al.	2013	GPL570	Japan	20	11.34	0.98	20	11.59	0.41	13	7	7	13
**GSE46408**	Yung-Ming Jeng, et al.	2013	GPL4133	China	6	12.06	1.14	6	13.02	0.48	5	0	1	6
**GSE50579**	Robert Geffers, et al.	2013	GPL14550	Germany	67	12.79	1.02	10	12.76	0.51	29	1	38	9
**GSE25599**	Biao-Yang Lin, et al.	2013	GPL9052	China	10	5.72	0.84	10	5.17	0.43	7	1	3	9
**GSE33294**	Chi-Ho Lin, et al.	2013	GPL16791	China	3	1.53	5.84	3	0.93	7.38	3	2	0	1
**GSE33006**	Yi Huang, et al.	2012	GPL570	China	3	11.59	0.10	3	10.13	2.21	3	1	0	2
**GSE31370**	Hyun Goo Woo, et al.	2012	GPL10558	South Korea	15	4.66	0.96	5	5.13	0.18	8	0	7	5
**GSE36376**	Cheol-Keun Park, et al.	2012	GPL10558	South Korea	240	10.75	0.83	193	10.81	0.38	53	9	187	184
**GSE36411**	Sang-Yeop Lee, et al.	2012	GPL10558	South Korea	42	12.37	0.92	42	12.61	0.37	19	8	23	34
**GSE29721**	Moshe Szyf, et al.	2011	GPL570	Canada	10	8.08	0.78	10	8.71	0.32	7	1	3	9
**GSE20140-GPL18461**	Yujin Hoshida, et al.	2011	GPL18461	USA	35	11.30	0.71	34	11.40	0.25	13	2	22	32
**GSE25097**	Chunsheng Zhang, et al	2011	GPL13369	USA	268	3.52	0.87	289	3.48	0.39	110	35	158	254
**GSE20140-GPL5474**	Yujin Hoshida, et al.	2011	GPL5474	USA	80	12.88	0.80	307	12.00	0.61	63	62	17	245
**GSE12941**	satow reiko, et al.	2010	GPL5175	Japan	10	10.35	0.59	10	10.36	0.22	4	0	6	10
**GSE14520-GPL571**	Xin-Wei Wang, et al.	2010	GPL571	USA	22	7.04	0.85	21	7.88	0.37	13	1	9	20
**GSE14520-GPL3921**	Xin-Wei Wang, et al.	2010	GPL3921	USA	225	7.57	0.95	220	8.17	0.52	114	27	111	193
**GSE19665**	Atsushi Kaneda, et al.	2010	GPL570	Japan	10	9.34	0.61	10	9.13	0.47	6	3	4	7
**GSE22058**	Julja Burchard, et al.	2010	GPL10687	USA	100	12.69	0.56	97	12.72	0.21	32	2	68	95
**GSE14323**	Kellie J. Archer, et al.	2009	GPL571	USA	55	8.24	0.44	60	8.47	0.60	49	40	6	20
**GSE17967**	Kellie J. Archer, et al.	2009	GPL571	USA	16	7.93	0.35	47	7.85	0.39	10	21	6	26
**GSE6222**	Ann-Ping Tsou, et al.	2008	GPL570	China	10	8.07	0.70	2	9.10	0.65	8	0	2	2
**GSE9839**	Luigi Terracciano, et al.	2008	GPL571	Switzerland	3	9.74	0.79	3	9.64	0.42	2	1	1	2
**GSE6764**	Elisa Wurmbach, et al.	2007	GPL570	USA	35	9.02	0.75	40	9.36	0.44	21	7	14	33

Supplementary Table 2. Clinical features of HCC patients based on the included datasets. HCC, hepatocellular carcinoma; N, number; M, mean; SD, standard deviation; AFP, Alpha-fetoprotein. To determine the statistical significance of difference between groups, independent-samples t-test or one-way analysis of variance (ANOVA) was used.

**Clinical features**	**N**	**Expression level of MAOA**			
		**M**	**SD**	**t or F**	**P-value**
**Tissue**					
HCC	344	12.4549	1.2029	2.2190	0.0270*
Non-cancer	33	12.9232	0.4574		
**Age（years）**					
<60	174	12.6178	1.0875	2.4660	0.0140*
≥60	169	12.3002	1.2869		
**Sex**					
Male	217	12.5002	1.2130	1.1340	0.2580
Female	94	12.3338	1.1784		
**Race**					
Asian	153	12.2688	1.3681	2.8830	0.0040*
Non-Asian	151	12.6664	1.0123		
**Ethnicity**					
Hispanic or Latino	18	12.7399	1.1187	0.9390	0.3595
Not Hispanic or Latino	340	12.4851	1.1810		
**Vital status**					
Alive	247	12.3685	1.2468	2.7430	0.0070*
Dead	64	12.7640	0.9631		
**Pathological stage**					
Stages I-II	240	12.4525	1.1501	-0.8340	0.4060
Stages III-IV	82	12.3135	1.3503		
**Tumor**					
T1-2	280	12.5206	1.0935	0.7615	0.4469
T3-4	94	12.4146	1.3687		
**Pathologic stage**					
Stage I	175	12.4424	1.0951	0.6345	0.5932
Stage II	87	12.5692	1.1467		
Stage III	86	12.3649	1.3448		
Stage IV	5	12.8406	0.3024		
**Tumor**					
T1	185	12.4696	1.0813	1.2426	0.2924
T2	95	12.6200	1.1160		
T3	81	12.3279	1.3935		
T4	13	12.9550	1.0982		
TX	1	12.8506	/		
**Node**					
Yes	4	12.4224	1.0359	-0.0321	0.9763
No	257	12.4392	1.2131		
**Metastasis**					

Yes	4	12.9285	0.2652	3.2193	0.0229*
No	270	12.4414	1.1979		
**Neoplasm histologic grade**					
G1	55	12.6491	0.9236	3.1157	0.0262*
G2	180	12.5732	1.1969		
G3	124	12.3655	1.1498		
G4	13	11.7081	1.4961		
**Child pugh classification grade**					
A	223	12.4861	1.1444	0.6112	0.5435
B	21	12.7682	0.7933		
C	1	12.4341	/		
**Adjacent hepatic tissue inflammation status**					
None	119	12.6330	0.9507	0.4732	0.6236
Mild	101	12.5451	1.3342		
Severe	19	12.3826	0.8483		
**AFP (ng/mL)**					
≤ 20	152	12.6351	0.9055	-2.5491	0.0113*
>20	132	12.2982	1.3082		
**Viral hepatitis**					
No	139	12.3702	1.3694	-1.1060	0.2700
Yes	189	12.5252	1.0791		
**Alcohol consumption**					
No	217	12.4472	1.2680	0.2680	0.7890
Yes	111	12.4834	1.0964		

Footnote: *A P-value < 0.05 indicates statistically significant.

Supplementary Table 3. Mutation types of MAOA in hepatocellular carcinoma patients based on the Catalogue of Somatic Mutations in Cancer.

Position	CDS Mutation	AA Mutation	Legacy Mutation ID	Count	Type
10	c.30G>T	p.A10=	COSM6251648	1	Substitution - coding silent
126	c.377A>G	p.N126S	COSM3766948	1	Substitution - Missense
129	c.385A>G	p.R129G	COSM4926748	1	Substitution - Missense
155	c.465G>T	p.M155I	COSM6333993	1	Substitution - Missense
220	c.660A>T	p.V220=	COSM5822820	1	Substitution - coding silent
232	c.695A>G	p.D232G	COSM6275505	1	Substitution - Missense
300	c.898A>G	p.M300V	COSM4950441	1	Substitution - Missense
327	c.980A>C	p.E327A	COSM131419	1	Substitution - Missense
331	c.991G>T	p.A331S	COSM6277430	1	Substitution - Missense
446	c.1338A>T	p.E446D	COSM4916798	1	Substitution - Missense

Supplementary Table 4. Disease ontology, gene ontology, and Kyoto Encyclopedia of Genes and Genomes analysis of the intersection of MAOA positively related co-expressed genes and downregulated differentially expressed genes.

ID	Description	Gene ratio	Bg ratio	P-value	Adjusted P	q-value	Gene ID	Count
**Disease Ontology**								
DOID:3393	coronary artery disease	34/301	348/8007	2.45E-07	0.000206	0.000181	CYP3A4, ARG1, LIPG, F11, CYP4F2, HP, CYP2C9, CYP2C8, LPA, ABCC9, APOA5, MBL2, APOC4, CYP1A2, CYP2C19, ESR1, IGF1, CD14, AGTR1, CNDP1, VKORC1, KCNMA1, CETP, HNF1A, ADAMTS13, VEGFA, ADRA2B, IGFBP3, PSMA6, TIMP3, CXCL12, CRP, NPC1L1, SELE	34
DOID:5844	myocardial infarction	27/301	279/8007	5.48E-06	0.002303	0.002029	ARG1, LIPG, F11, CYP4F2, HP, CYP2C9, CYP2C8, LPA, ABCC9, APOA5, MBL2, CYP1A2, CYP2C19, ESR1, IGF1, CD14, AGTR1, KCNMA1, CETP, ADAMTS13, VEGFA, ADRA2B, PSMA6, TIMP3, CXCL12, CRP, SELE	27
DOID:655	inherited metabolic disorder	29/301	331/8007	1.7E-05	0.004765	0.004199	BHMT, NR1I2, ABCG5, CBS, LPA, XDH, ABCC9, APOA5, BCKDHA, GCKR, CYP2E1, CYP1A2, ESR1, IGF1, ASL, LCAT, AGTR1, XBP1, PNPLA3, CETP, VEGFA, ECM1, PLIN1, HBB, CXCL12, HAMP, CRP, NPC1L1, SELE	29
DOID:13580	cholestasis	10/301	62/8007	9.19E-05	0.015759	0.013886	ABCB11, NR1I3, NR1I2, MME, SLC22A1, ESR1, CD14, SLC22A3, VEGFA, SELE	10
DOID:18	urinary system disease	35/301	478/8007	0.000102	0.015759	0.013886	CYP4A11, ACACB, HP, CYP2C9, MME, LPA, XDH, ITIH4, ADRA1A, MBL2, ESR1, IGF1, ALPL, PROZ, ADRA1B, AGTR1, CRHBP, DUSP1, CETP, MT2A, ICAM3, ADAMTS13, CCL15, PTH1R, VEGFA, NGFR, SLC34A1, TRPV1, IGFBP3, HAMP, CRP, FOS, ILK, MT1A, SELE	35
**Biological Process**								
GO:0016054	organic acid catabolic process	45/515	250/17913	2.95E-23	5.92E-20	5.31E-20	ACADL, MAT1A, AGXT, HPD, ALDH4A1, CYP4F3, ADHFE1, ARG1, AMACR, CBS, AGXT2, CYP4F2, TAT, CYP4A11, CYP4F12, AASS, ACACB, AKR1D1, AMDHD1, CTH, HAO2, ACADS, BCKDHA, CYP39A1, GSTZ1, TDO2, FTCD, ALDH8A1, KMO, SDS, AADAT, MTHFS, ASPA, CSAD, GLDC, HOGA1, CYP26A1,	45

							PLIN5, HAL, UROC1, IDO2, LYVE1, CEL, STAB2, PRODH	
GO:0046395	carboxylic acid catabolic process	45/515	250/17913	2.95E-23	5.92E-20	5.31E-20	ACADL, MAT1A, AGXT, HPD, ALDH4A1, CYP4F3, ADHFE1, ARG1, AMACR, CBS, AGXT2, CYP4F2, TAT, CYP4A11, CYP4F12, AASS, ACACB, AKR1D1, AMDHD1, CTH, HAO2, ACADS, BCKDHA, CYP39A1, GSTZ1, TDO2, FTCD, ALDH8A1, KMO, SDS, AADAT, MTHFS, ASPA, CSAD, GLDC, HOGA1, CYP26A1, PLIN5, HAL, UROC1, IDO2, LYVE1, CEL, STAB2, PRODH	45
GO:1901605	alpha-amino acid metabolic process	38/515	200/17913	1.17E-20	1.56E-17	1.4E-17	BHMT, CPS1, GLYAT, MAT1A, AGXT, HPD, ALDH4A1, GNMT, ADHFE1, ARG1, CBS, AGXT2, TAT, AASS, AMDHD1, CTH, NAGS, GSTZ1, TDO2, FTCD, ALDH8A1, KMO, SDS, AADAT, MTHFS, ASPA, CSAD, GLDC, HOGA1, DCT, HAL, UROC1, IDO2, MTHFD2L, ADSSL1, OAT, ACCS, PRODH	38
GO:0044282	small molecule catabolic process	52/515	404/17913	7.75E-20	7.78E-17	6.98E-17	ACADL, MAT1A, AGXT, HPD, ALDH4A1, CYP4F3, ADHFE1, ARG1, AMACR, CBS, AGXT2, CYP4F2, TAT, CYP4A11, CYP4F12, AASS, ACACB, AKR1D1, AMDHD1, CTH, HAO2, ACADS, BCKDHA, ADH4, CYP1A1, CYP39A1, GSTZ1, TDO2, FTCD, ALDH8A1, KMO, SDS, AADAT, MTHFS, SULT1B1, SULT1E1, ASPA, CSAD, CDA, GLDC, HOGA1, CYP26A1, PLIN5, HAL, UROC1, ENO3, IDO2, LYVE1, CYP46A1, CEL, STAB2, PRODH	52
GO:0006520	cellular amino acid metabolic process	47/515	347/17913	6.31E-19	5.07E-16	4.54E-16	BHMT, CPS1, GLYAT, MAT1A, AGXT, HPD, ALDH4A1, GNMT, ADHFE1, ARG1, CBS, AGXT2, TAT, AASS, PSMA2, AMDHD1, CTH, BCKDHA, NAGS, GSTZ1, TDO2, FTCD, ALDH8A1, KMO, SDS, AADAT, MTHFS, ACY1, ASPA, CSAD, GLS2, SLC7A2, GLDC, HOGA1, SCLY, DCT, HAL, UROC1, IDO2, PSMC1, ASPG, MTHFD2L, PSMA6, ADSSL1, OAT, ACCS, PRODH	47

Cellular Component

GO:0072562	blood microparticle	18/539	147/18678	2.51E-07	0.00011	0.000106	C8A, AFM, HP, ITIH4, CFB, C1R, CFHR3, A1BG, ORM1, SERPINA3, FCN2, C9, CD5L, ORM2, FCN3, HBB, PZP, HBD	18
GO:0034358	plasma lipoprotein particle	7/539	37/18678	7.78E-05	0.008693	0.00836	LPA, APOA5, APOC4, APOF, LCAT, SAA4, CETP	7
GO:1990777	lipoprotein particle	7/539	37/18678	7.78E-05	0.008693	0.00836	LPA, APOA5, APOC4, APOF, LCAT, SAA4, CETP	7
GO:0034364	high-density lipoprotein particle	6/539	26/18678	7.9E-05	0.008693	0.00836	APOA5, APOC4, APOF, LCAT, SAA4, CETP	6
GO:0032994	protein-lipid complex	7/539	39/18678	0.000111	0.009727	0.009355	LPA, APOA5, APOC4, APOF, LCAT, SAA4, CETP	7
**Molecular Function**								
GO:0004497	monooxygenase activity	23/511	86/16969	5.11E-16	3.25E-13	2.79E-13	CYP3A4, CYP4F3, CYP4F2, CYP4A11, CYP4F12, CYP2C9, CYP2C8, CYP3A43, CYP1A1, CYP39A1, CYP2E1, CYP1A2, CYP2C19, CYP2A6, CYP2B6, KMO, CYP2A7, CYP26A1, CYP4A22, DBH, CYP46A1, CYP11B1, MICAL3	23
GO:0048037	cofactor binding	49/511	438/16969	1.86E-15	5.91E-13	5.08E-13	ACADL, CYP3A4, AGXT, CYP4F3, CBS, AGXT2, CYP4F2, OGDHL, TAT, CYP4A11, CYP4F12, ACACB, CTH, XDH, ACADS, CYP3A43, ADH4, CYP1A1, CYP39A1, CYP2E1, CYP1A2, CYP2C19, CYP2A6, CYP2B6, NDUFV2, TDO2, GPD1, KMO, SDS, AADAT, GCH1, CSAD, DHODH, VKORC1, GLDC, CYP26A1, HPGD, PNPLA3, IDO2, CYP4A22, DBH, CYP46A1, AOC2, CYP11B1, STEAP4, ACCS, GSTM2, MICAL3, PRODH	49
GO:0020037	heme binding	21/511	106/16969	5.58E-12	1.18E-09	1.01E-09	CYP3A4, CYP4F3, CBS, CYP4F2, CYP4A11, CYP4F12, CYP3A43, CYP1A1, CYP39A1, CYP2E1, CYP1A2, CYP2C19, CYP2A6, CYP2B6, TDO2, CYP26A1, IDO2, CYP4A22, CYP46A1, CYP11B1, STEAP4	21
GO:0046906	tetrapyrrole binding	21/511	116/16969	3.41E-11	5.41E-09	4.65E-09	CYP3A4, CYP4F3, CBS, CYP4F2, CYP4A11, CYP4F12, CYP3A43, CYP1A1, CYP39A1, CYP2E1, CYP1A2,	21

							CYP2C19, CYP2A6, CYP2B6, TDO2, CYP26A1, IDO2, CYP4A22, CYP46A1, CYP11B1, STEAP4	
GO:0005506	iron ion binding	23/511	144/16969	5.66E-11	7.19E-09	6.17E-09	CYP3A4, CYP8B1, CYP4F3, CYP4F2, CYP4A11, CYP4F12, CYP2C9, CYP2C8, XDH, CYP3A43, CYP1A1, CYP39A1, BBOX1, CYP2E1, CYP1A2, CYP2C19, CYP2A6, CYP2B6, CYP2A7, CYP26A1, CYP4A22, CYP46A1, CYP11B1	23
**KEGG pathway**								
hsa00830	Retinol metabolism	15/325	67/8011	4.9E-08	1.34E-05	1.21E-05	CYP3A4,ADH1A,CYP4A11,RDH16,CYP2C9,CYP2C8,ADH4,CYP1A1,CYP1A2,CYP2A6,CYP2B6,RDH5,CYP26A1,CYP4A22,UGT2B17	15
hsa05204	Chemical carcinogenesis	16/325	82/8011	1.36E-07	1.87E-05	1.68E-05	CYP3A4,ADH1A,CYP2C9,CYP2C8,CYP3A43,ADH4,CYP1A1,NAT2,CYP2E1,CYP1A2,CYP2C19,CYP2A6,GSTA5,UGT2B17,GSTT2,GSTM2	16
hsa00982	Drug metabolism - cytochrome P450	14/325	72/8011	8.76E-07	8E-05	7.19E-05	CYP3A4,ADH1A,CYP2C9,CYP2C8,ADH4,CYP2E1,CYP1A2,CYP2C19,CYP2A6,CYP2B6,GSTA5,UGT2B17,GSTT2,GSTM2	14
hsa00980	Metabolism of xenobiotics by cytochrome P450	13/325	76/8011	9.61E-06	0.000658	0.000592	CYP3A4,ADH1A,CYP2C9,ADH4,CYP1A1,CYP2E1,CYP1A2,CYP2A6,CYP2B6,GSTA5,UGT2B17,GSTT2,GSTM2	13
hsa00140	Steroid hormone biosynthesis	11/325	60/8011	2.38E-05	0.001093	0.000982	CYP3A4,AKR1D1,CYP1A1,CYP2E1,SRD5A2,CYP1A2,SULT1E1,HSD3B2,HSD17B3,UGT2B17,CYP11B1	11

Supplementary Table 5. Disease ontology, gene ontology, and Kyoto Encyclopedia of Genes and Genomes analysis of the intersection of MAOA negatively related co-expressed genes and upregulated differentially expressed genes.

ID	Description	Gene ratio	Bg ratio	P-value	Adjusted P	q-value	Gene ID	Count
**Disease Ontology**								
DOID:12155	lymphocytic choriomeningitis	3/93	8/8007	8.14E-05	0.031742	0.026821	CCR5,CD86,CXCR3	3
DOID:2377	multiple sclerosis	9/93	169/8007	0.000141	0.031742	0.026821	BDKRB1,CCL5,CCR5,CD1A,CHIT1,CXCR3,HLA-DPB1,HLA-DRA,SLC1A3	9
DOID:3213	demyelinating disease	9/93	175/8007	0.000184	0.031742	0.026821	BDKRB1,CCL5,CCR5,CD1A,CHIT1,CXCR3,HLA-DPB1,HLA-DRA,SLC1A3	9
DOID:6367	acral lentiginous melanoma	2/93	3/8007	0.000397	0.051276	0.043326	CDKN2A,CSPG4	2
DOID:14268	sclerosing cholangitis	3/93	15/8007	0.000624	0.051276	0.043326	CCL5,CCR5,CDKN2A	3
**Biological Process**								
GO:0042110	T cell activation	18/158	443/17913	7.60E-08	0.000192	0.000173	AIF1,CCL5,CD2,CD3D,CD3G,CD74,CD83,CD86,FOXJ1,HLA-DMB,HLA-DOA,HLA-DPA1,HLA-DPB1,PLA2G2D,RELB,SIT1,TNFRSF4,ZNF683	18
GO:0051249	regulation of lymphocyte activation	16/158	401/17913	5.35E-07	0.000676	0.00061	AIF1,CCL5,CD2,CD74,CD83,CD86,FOXJ1,HLA-DMB,HLA-DOA,HLA-DPA1,HLA-DPB1,IL27RA,SIT1,SOX11, TNFRSF4,ZNF683	16
GO:0002694	regulation of leukocyte activation	17/158	492/17913	1.70E-06	0.001429	0.001288	AIF1,C1QA,CCL5,CD2,CD74,CD83,CD86,FOXJ1,HLA-DMB,HLA-DOA,HLA-DPA1,HLA-DPB1,IL27RA,SIT1, SOX11,TNFRSF4,ZNF683	17
GO:0050863	regulation of T cell activation	13/158	307/17913	3.42E-06	0.002162	0.001949	AIF1,CCL5,CD2,CD74,CD83,CD86,FOXJ1,HLA-DMB,HLA-DOA,HLA-DPA1,HLA-DPB1,SIT1,ZNF683	13
GO:0070098	chemokine-mediated	6/158	51/17913	5.56E-06	0.002808	0.002531	CCL26,CCL5,CCR5,CCR8,CXCR3,ROBO1	6

	signaling pathway							
**Cellular Component**								
GO:0042613	MHC class II protein complex	7/163	16/18678	3.63E-11	8.78E-09	7.79E-09	CD74,HLA-DMB,HLA-DOA,HLA-DPA1,HLA-DPB1,HLA-DQB2,HLA-DRA	7
GO:0042611	MHC protein complex	7/163	25/18678	1.43E-09	1.73E-07	1.53E-07	CD74,HLA-DMB,HLA-DOA,HLA-DPA1,HLA-DPB1,HLA-DQB2,HLA-DRA	7
GO:0030669	clathrin-coated endocytic vesicle membrane	6/163	40/18678	1.21E-06	9.76E-05	8.66E-05	CD74,FZD2,HLA-DPA1,HLA-DPB1,HLA-DQB2,HLA-DRA	6
GO:0071556	integral component of lumenal side of endoplasmic reticulum membrane	5/163	29/18678	4.77E-06	0.00022	0.000195	CD74,HLA-DPA1,HLA-DPB1,HLA-DQB2,HLA-DRA	5
GO:0098553	lumenal side of endoplasmic reticulum membrane	5/163	29/18678	4.77E-06	0.00022	0.000195	CD74,HLA-DPA1,HLA-DPB1,HLA-DQB2,HLA-DRA	5
**Molecular Function**								
GO:0032395	MHC class II receptor activity	4/154	10/16969	1.31E-06	0.000376	0.000334	HLA-DOA,HLA-DPA1,HLA-DQB2,HLA-DRA	4
GO:0023026	MHC class II protein complex binding	4/154	16/16969	1.09E-05	0.00156	0.001389	CD74,HLA-DMB,HLA-DOA,HLA-DRA	4
GO:0015026	coreceptor activity	5/154	40/16969	2.94E-05	0.0028	0.002494	CCR5,CCR8,CD86,IGSF1,RAMP2	5

GO:0023023	MHC protein complex binding	4/154	25/16969	7.11E-05	0.005086	0.00453	CD74,HLA-DMB,HLA-DOA,HLA-DRA	4
GO:0042277	peptide binding	8/154	215/16969	0.000782	0.041215	0.03671	BDKRB1,C1QA,CD74,HLA-DPA1,HLA-DPB1,HLA-DRA,MSR1,RAMP2	8
**KEGG pathway**								
hsa05323	Rheumatoid arthritis	11/79	93/7946	1.45E-09	2.25E-07	1.76E-07	ACP5,ATP6V0D2,CCL20,CCL5,CD86,HLA-DMB,HLA-DOA,HLA-DPA1,HLA-DPB1,HLA-DRA,LTB	11
hsa04640	Hematopoietic cell lineage	10/79	99/7946	4.02E-08	3.12E-06	2.43E-06	CD1A,CD2,CD3D,CD3G,CD5,HLA-DMB,HLA-DOA,HLA-DPA1,HLA-DPB1,HLA-DRA	10
hsa05166	Human T-cell leukemia virus 1 infection	13/79	219/7946	1.96E-07	1.01E-05	7.92E-06	BUB1B,CD3D,CD3G,CDKN2A,E2F1,E2F2,HLA-DMB,HLA-DOA,HLA-DPA1,HLA-DPB1,HLA-DRA,MSX1, RELB	13
hsa05150	Staphylococcus aureus infection	9/79	96/7946	3.85E-07	1.49E-05	1.16E-05	C1QA,C1QC,C3AR1,FPR3,HLA-DMB,HLA-DOA,HLA-DPA1,HLA-DPB1,HLA-DRA	9
hsa05330	Allograft rejection	6/79	38/7946	1.71E-06	5.29E-05	4.13E-05	CD86,HLA-DMB,HLA-DOA,HLA-DPA1,HLA-DPB1,HLA-DRA	6

Supplementary Table 6. Clinical significance of MAOA protein expression level based on in-house tissue microarray.

Clinical features	MAOA protein expression level			t	df	P-value
	N	M	SD			
**Tissue**						
HCC	315	8.4190	2.4886	18.3753	495.1975	<0.0001*
non-HCC	192	11.5208	1.3023			
**Age**						
<60	231	8.5714	2.4782	1.8087	313.0000	0.0715
>=60	84	8.0000	2.4837			
**Gender**						
Male	274	8.4672	2.5651	1.1064	64.2379	0.2727
Female	41	8.0976	1.8948			
**Pathological stage**						
I	17	8.7059	2.1144	0.1547	312.0000	0.9608
I-II	25	8.1600	2.4440			
II	204	8.4314	2.4735			
II-III	27	8.5926	2.6495			
III	40	8.4000	2.6871			
**TNM**						
T1-T2	235	9.0894	2.0351	8.0882	109.9644	<0.0001*
T3-T4	79	6.4304	2.6732			
**TNM_T**						
T1	3	12.0000	0.0000	30.5695	313.0000	<0.0001*
T2	232	9.0517	2.0209			
T3	72	6.4444	2.7316			
T4	7	6.2857	2.1381			
**TNM_N**						
N0	307	8.4300	2.4992	0.4507	312.0000	0.6525
N1	7	8.0000	2.3094			
**TNM_M**						
M0	313	8.4345	2.4839	1.7825	312.0000	0.0756
M1	1	4.0000	/			
**Smoking**						
No	91	8.2198	2.6907	0.3919	159.0000	0.6956
Yes	70	8.0571	2.5015			
**Alcohol consumption**						
No	82	8.3415	2.6815	0.9551	159.0000	0.3410
Yes	79	7.9494	2.5212			
**Hepatitis history**						
No	60	8.4000	2.7259	0.7184	147.0000	0.4736
Yes	89	8.0899	2.4847			
**Family liver cancer history**						
No	132	8.3939	2.5615	-0.0574	139.0000	0.9543

Yes	9	8.4444	2.4037			
**Tumor size (cm)**						
<3	10	9.6000	2.7968	1.7131	133.0000	0.0890
>=3	125	8.0960	2.6622			

Footnote: *A P-value < 0.05 indicates statistical significance.

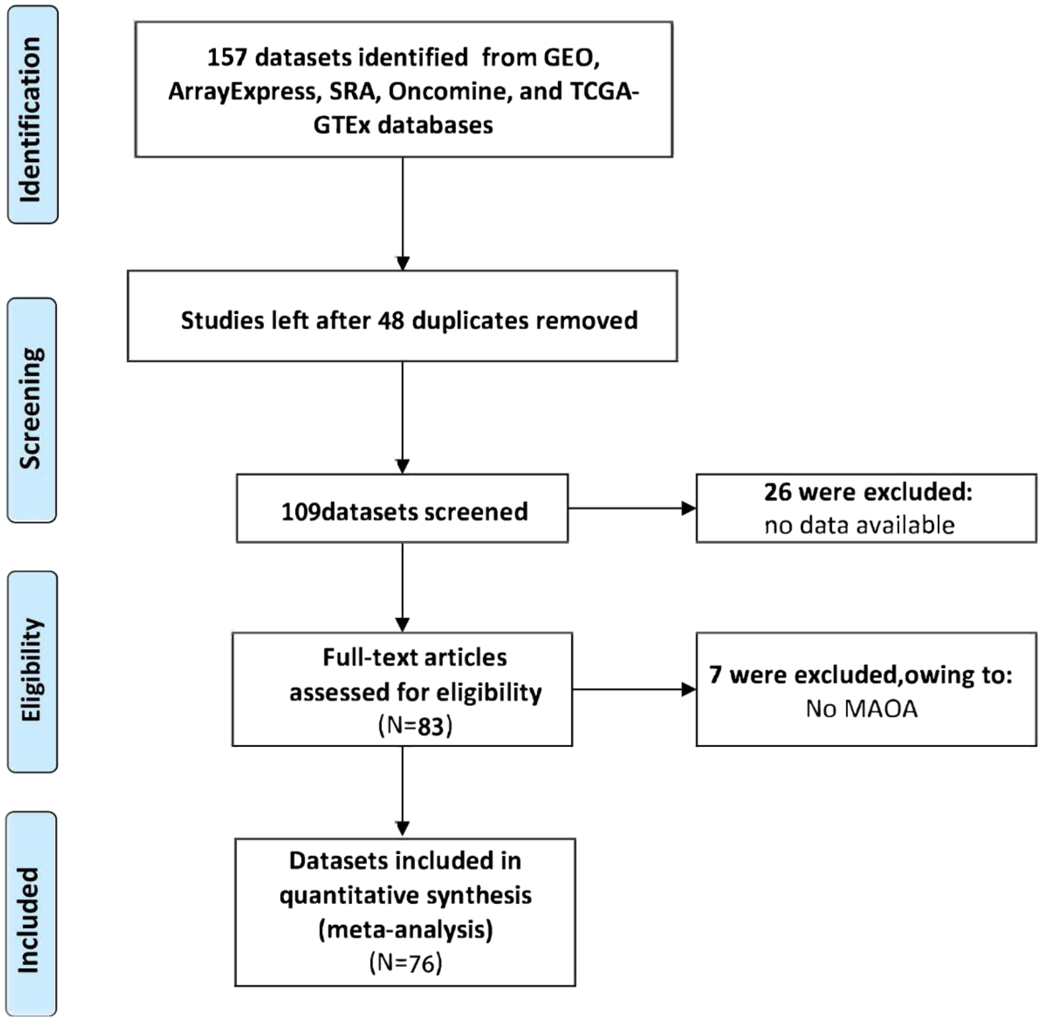

Supplementary Figure 1. The flow chart of the included datasets for differentially expressed gene analysis.

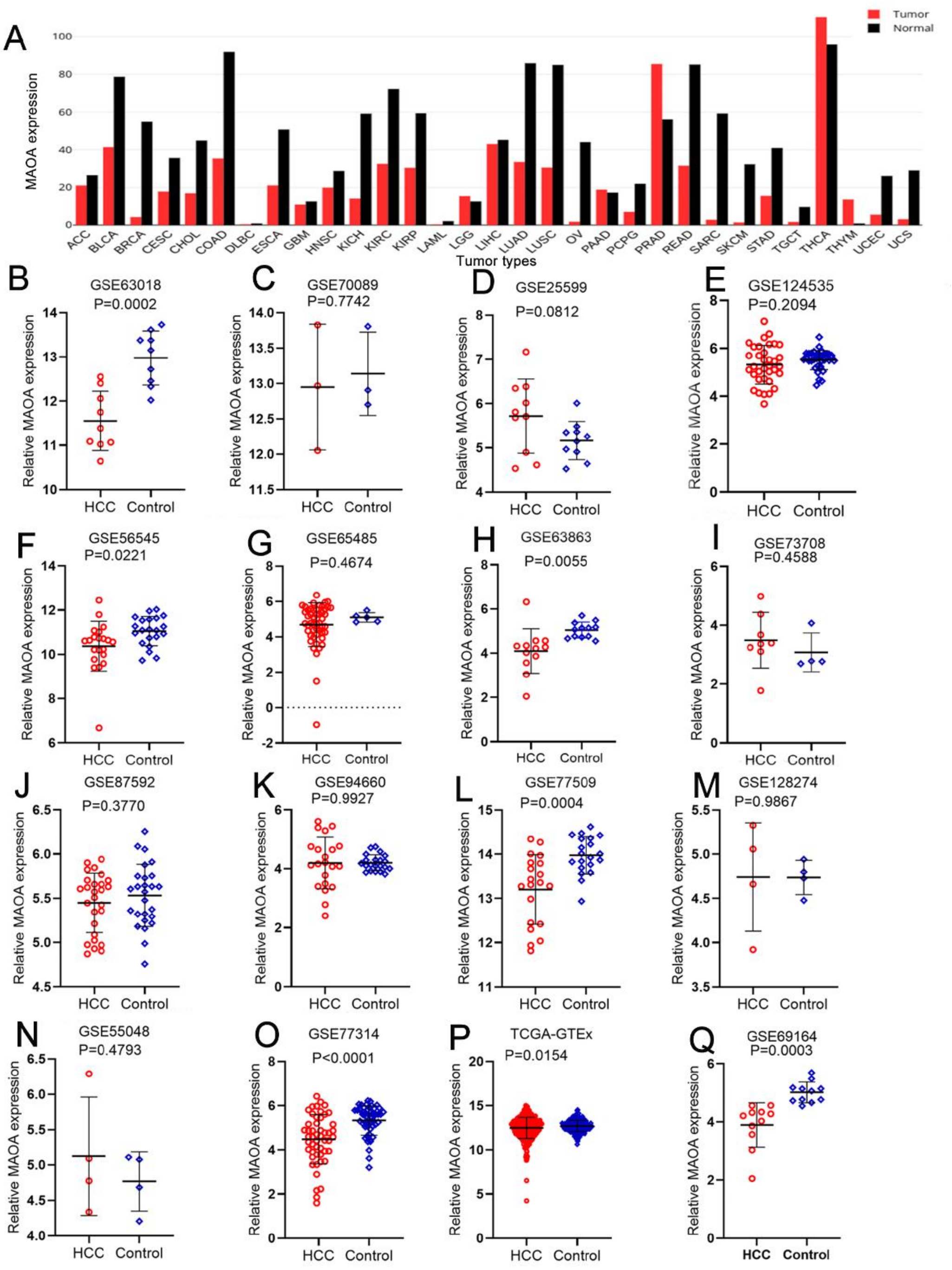

Supplementary Figure 2. The expression status of *MAOA* in various tumors and scatter plot of the *MAOA* expression levels. Decreased *MAOA* expression levels were found in hepatocellular carcinoma patients compared to non-cancer groups based on the included RNA sequencing datasets. TCGA, the Cancer Genome Atlas; GTEx, the Genotype-Tissue Expression; LIHC, liver hepatocellular carcinoma. The error bars in the scatter dot plot line at mean with standard deviation.

(A) The expression statuses in 31 kinds of tumors from Gene Expression Profiling Interactive Analysis (GEPIA). **(B)** GSE63018, **(C)** GSE70089, **(D)** GSE25599, **(E)** GSE124535, **(F)** GSE56545, **(G)** GSE65485, **(H)** GSE63863, **(I)** GSE73708, **(J)** GSE87592, **(K)** GSE94660, **(L)** GSE77509, **(M)** GSE128274, **(N)**GSE55048, **(O)** GSE77314, **(P)** TCGA-GTEx, and **(Q)** GSE69164.

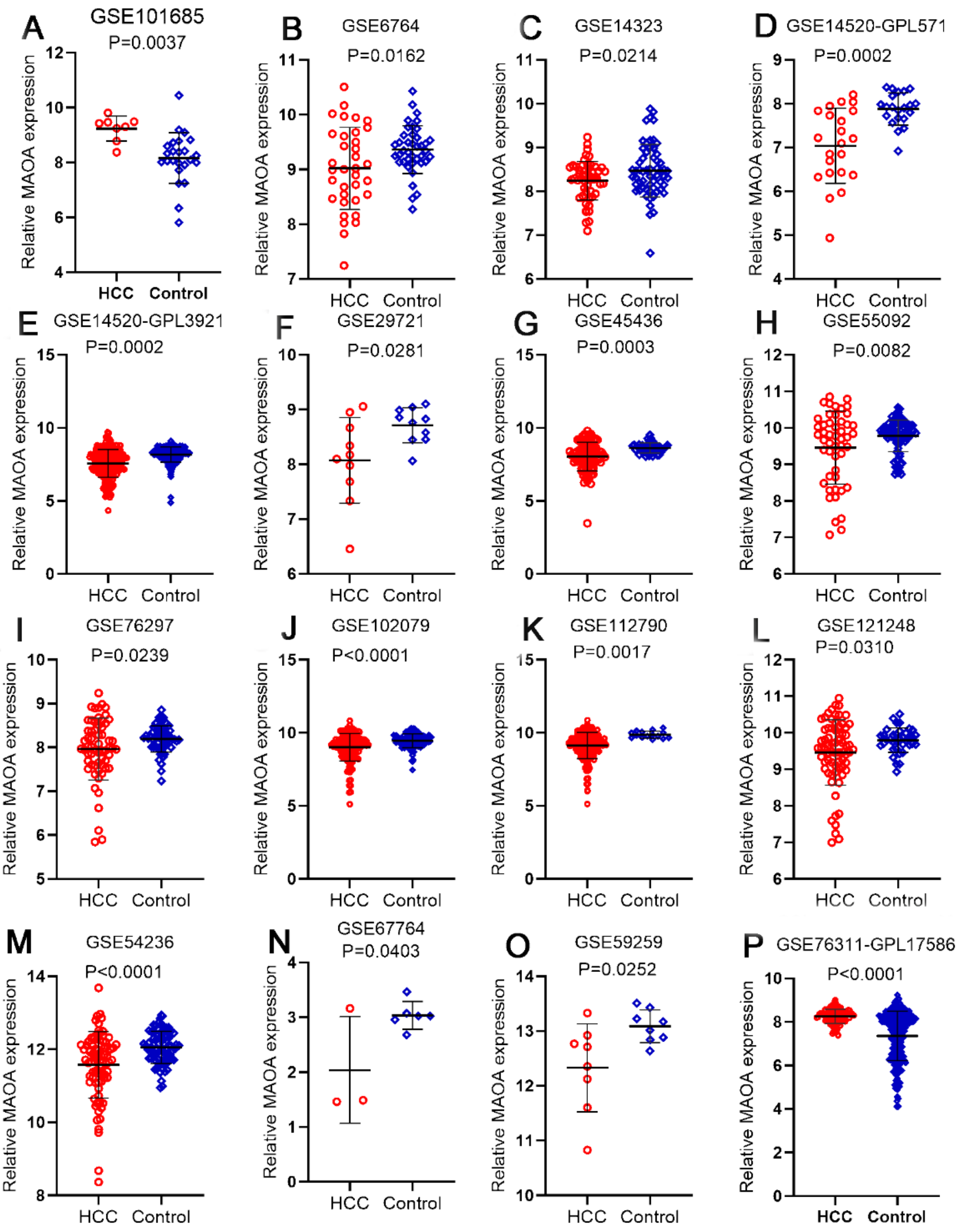

Supplementary Figure 3. Scatter plot of the MAOA expression between hepatocellular carcinoma patients and non-cancer groups based on the included genechip datasets. The error bars in the scatter dot plot line at mean with standard deviation.

(A) GSE101685, **(B)** GSE6764, **(C)** GSE14323, **(D)** GSE14520-GPL571, **(E)** GSE14520-GPL3921, **(F)** GSE29721, **(G)** GSE45436, **(H)** GSE55092, **(I)** GSE76297, **(J)** GSE102079, **(K)** GSE112790, **(L)** GSE121248, **(M)** GSE54236, **(N)** GSE67764, **(O)** GSE59259, and **(P)** GSE76311-GPL17586.

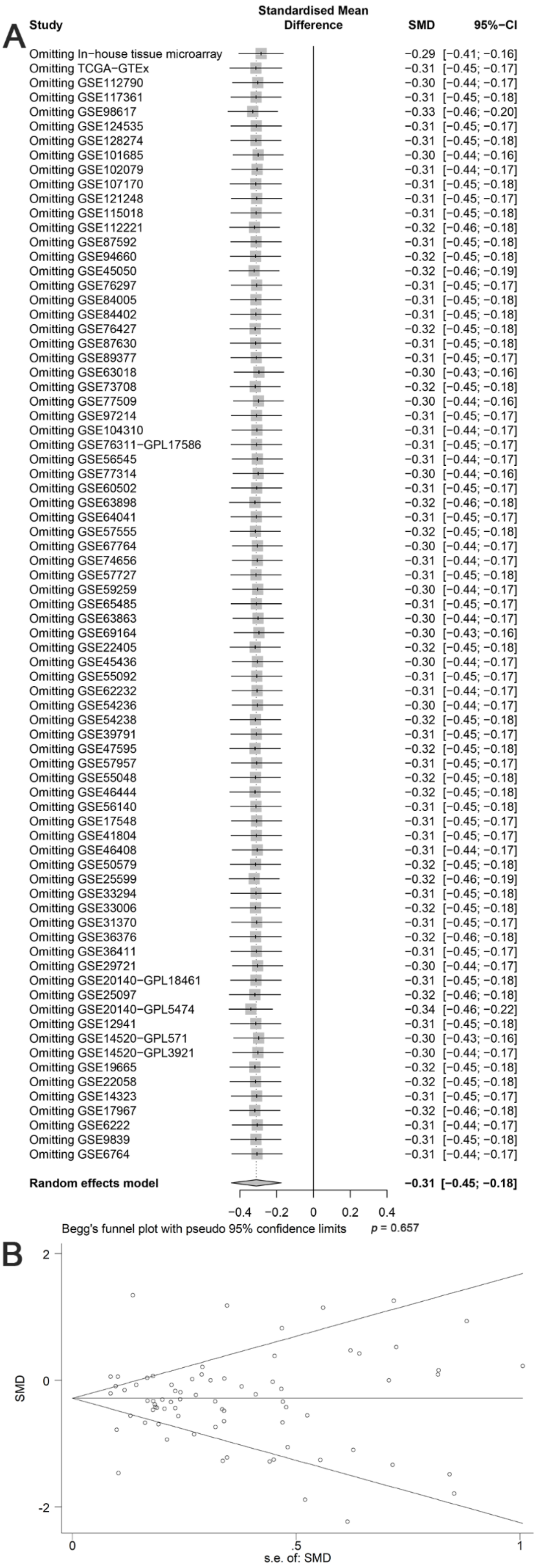

Supplementary Figure 4. Assessment of the heterogeneity source. **(A)** Sensitive analysis indicated that no significant difference existed among the enrolled studies. **(B)** Begg's funnel plot showed that there was no significant publication bias.

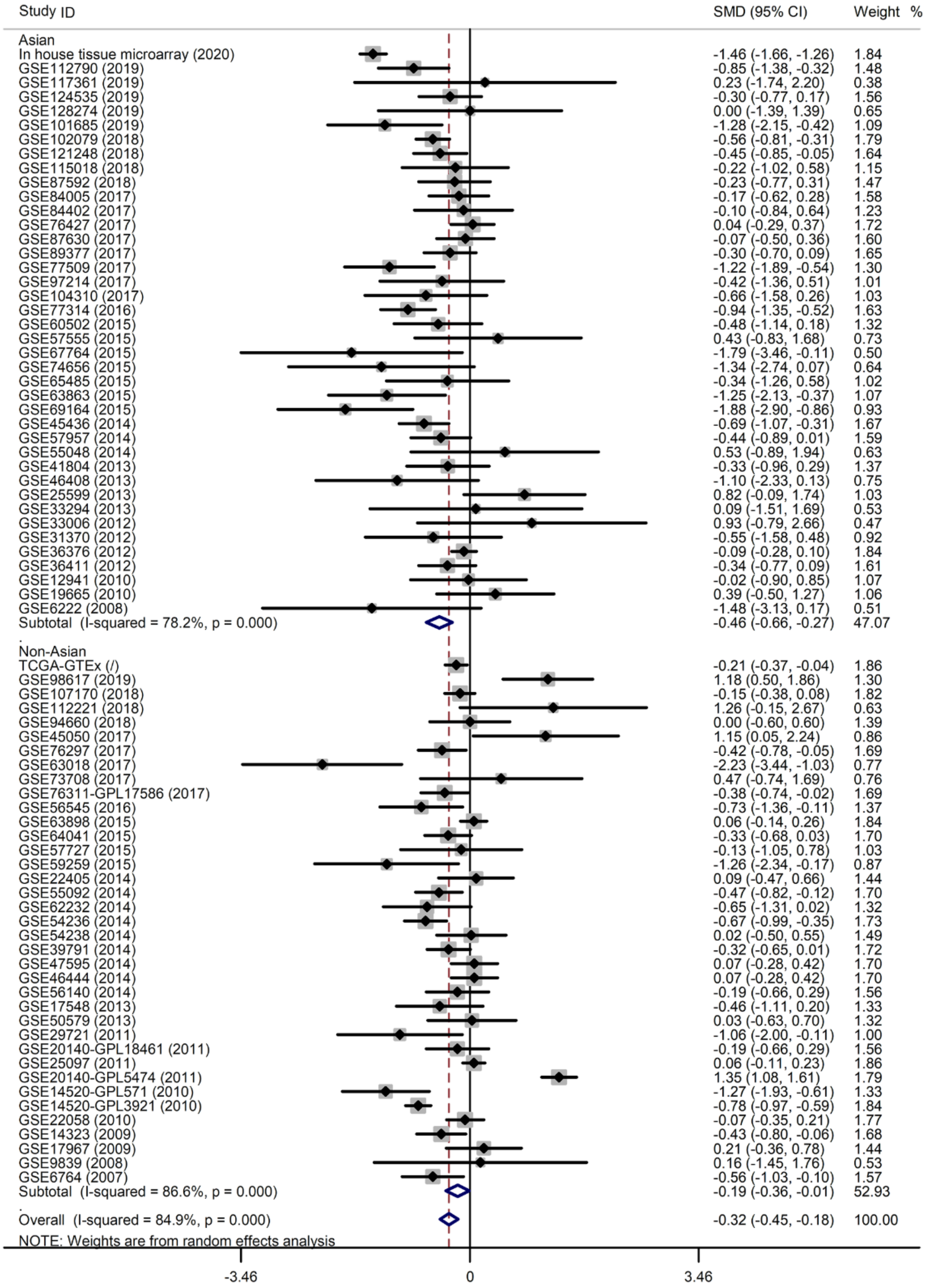

Supplementary Figure 5. Subgroup analysis based on the ethnicities of hepatocellular carcinoma patients. No significant difference between subgroups was found.

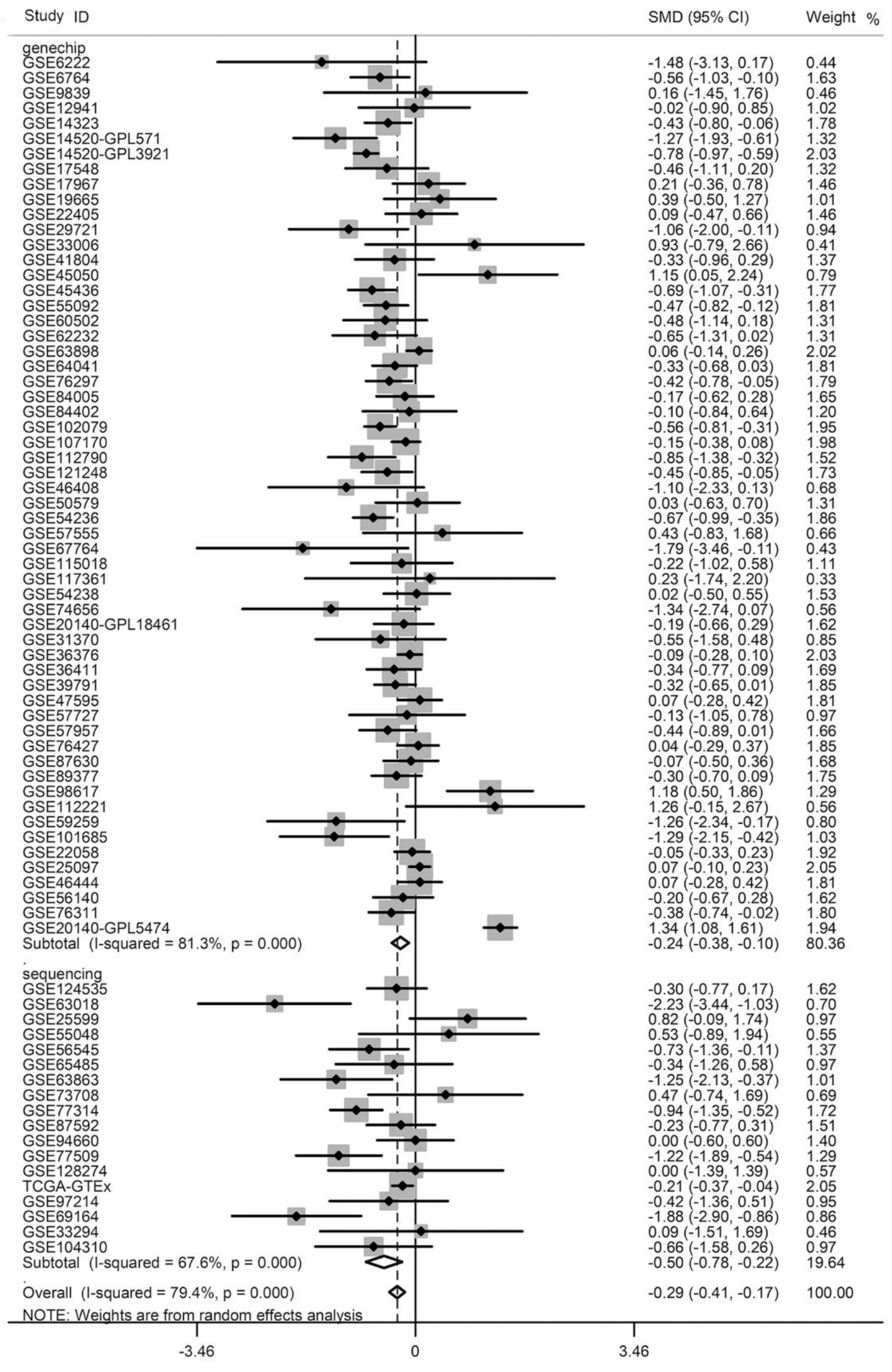

Supplementary Figure 6. Subgroup analysis based on the included datasets in terms of the methods used to determine the expression levels of *MAOA* in hepatocellular carcinoma patients (i.e., genechip and RNA-sequencing). No significant difference between subgroups was found.

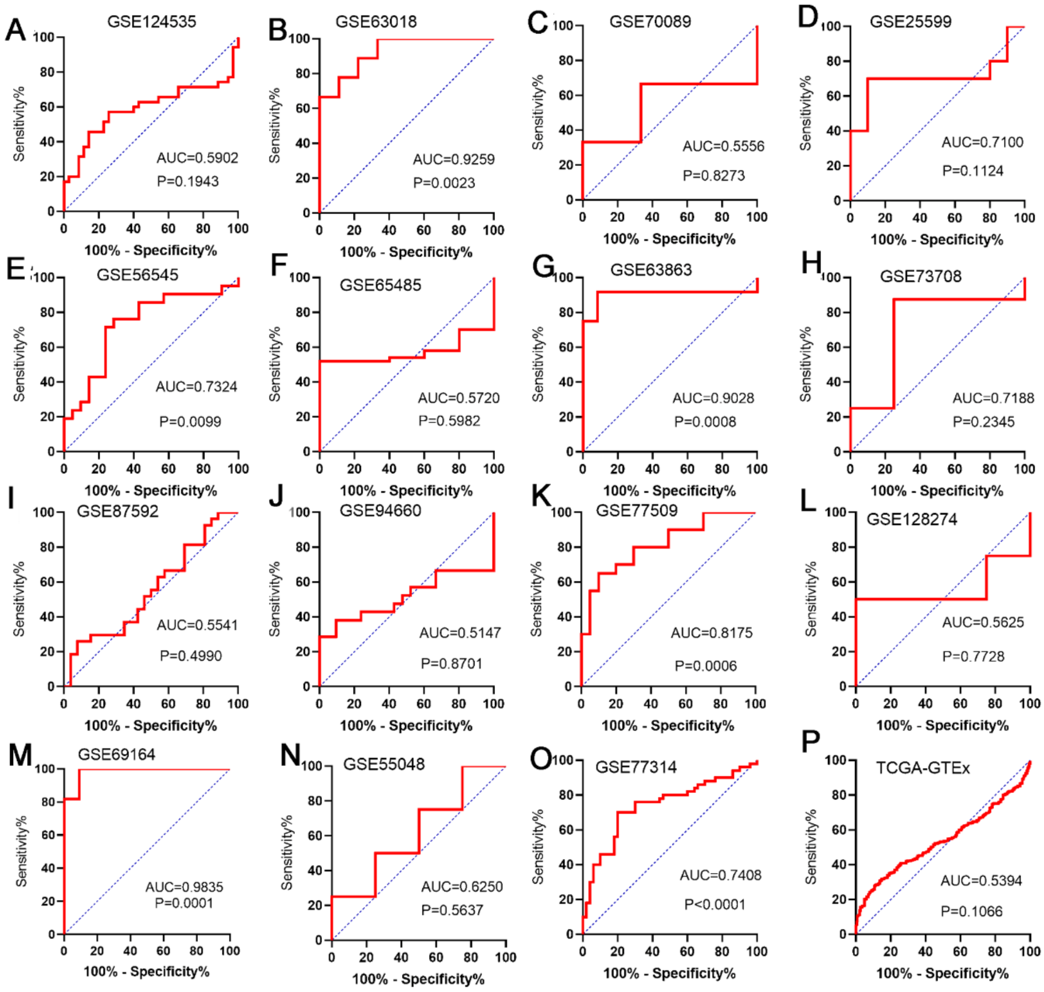

Supplementary Figure 7. The Receiver Operating Characteristic (ROC) curve of *MAOA* expression based on the included RNA sequencing datasets. TCGA, the Cancer Genome Atlas; GTEx, the Genotype-Tissue Expression.
(A) GSE124535, **(B)** GSE63018, **(C)** GSE70089, **(D)** GSE25599, **(E)** GSE56545, **(F)** GSE65485, **(G)** GSE63863, **H**） GSE73708,**(I)** GSE87592, **(J)** GSE94660,(K) GSE77509, **(L)** GSE128274, **(M)** GSE69164, **(N)** GSE55048, **(O)** GSE77314, and **(P)** TCGA-GTEx.

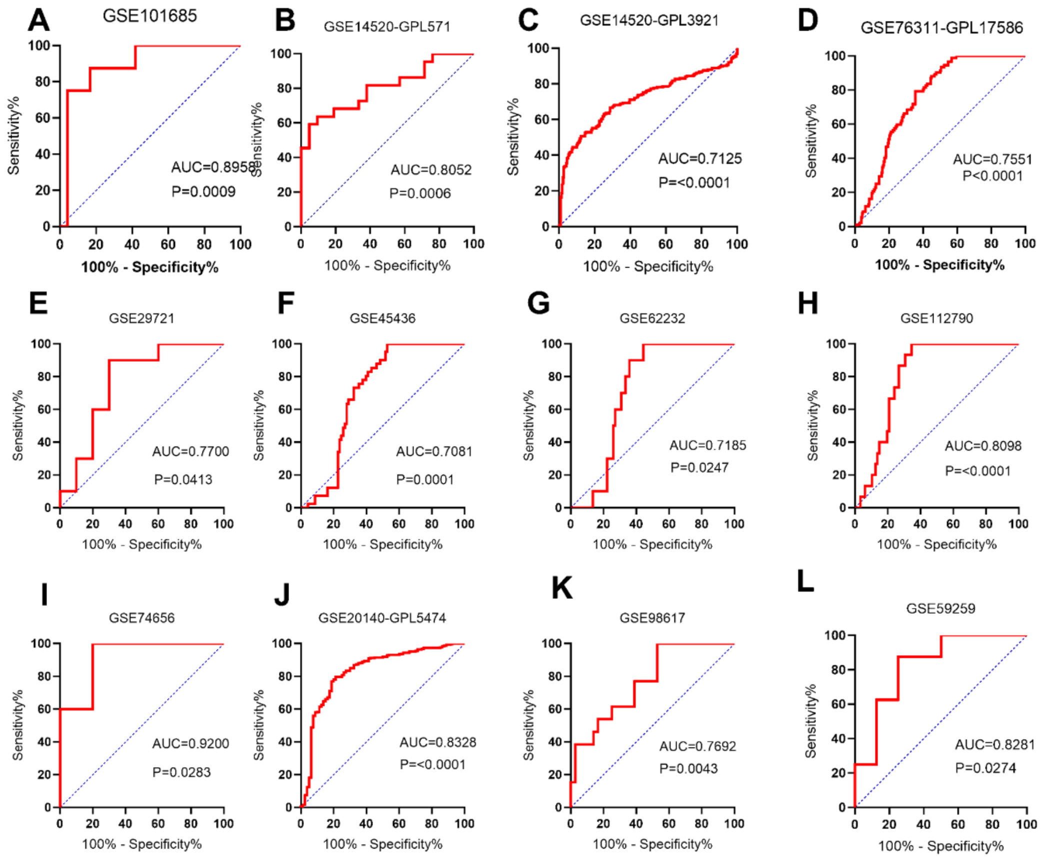

Supplementary Figure 8. The Receiver Operating Characteristic (ROC) curve of MAOA expression based on the included genechip datasets.

(A) GSE101685, **(B)** GSE14520-GPL571, **(C)** GSE14520-GPL3921, **(D)** GSE76311-GPL17586, **(E)** GSE29721, **(F)** GSE45436, **(G)** GSE62232, **(H)** GSE112790, **(I)** GSE74656, **(J)** GSE20140-GPL5474, **(K)** GSE98617, and **(L)** GSE59259.

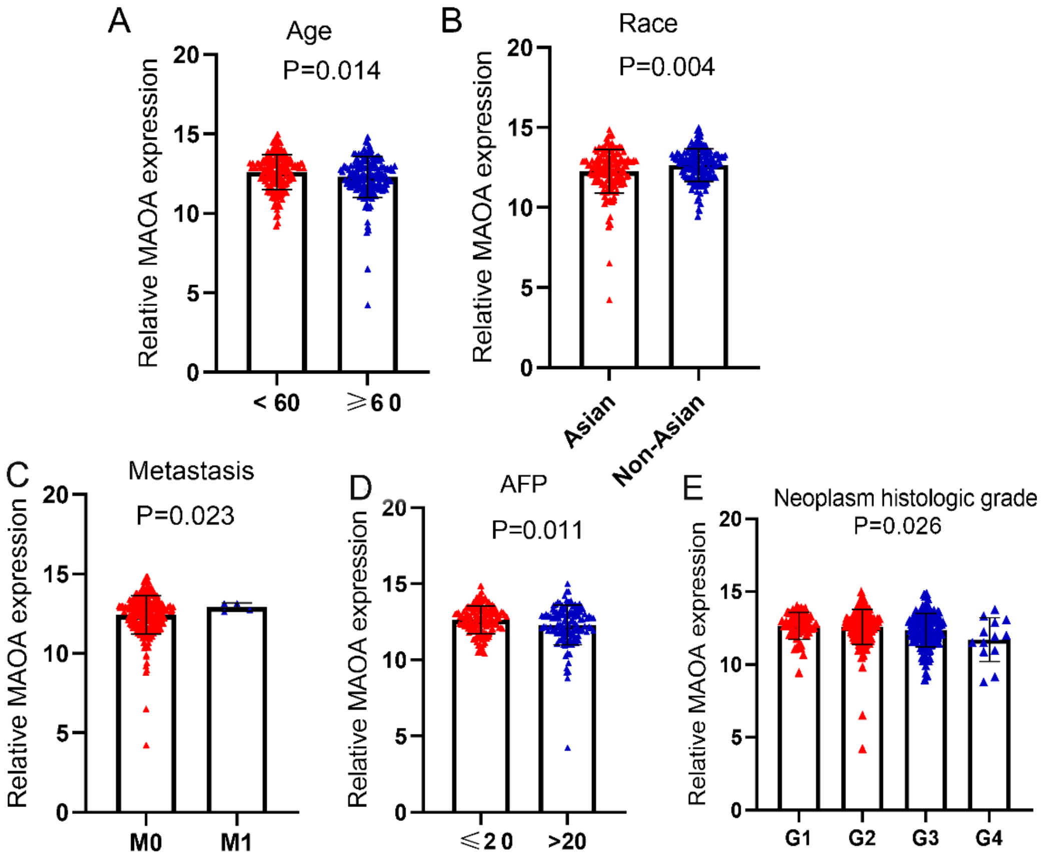

Supplementary Figure 9. The relationships between the clinical features of HCC patients and *MAOA* mRNA expression level. The down-regulated expression level of *MAOA* was relevant to age, race, tumor metastasis status, neoplasm histologic grade, and AFP concentration (> 20 ng/mL) (all with P-values < 0.05).

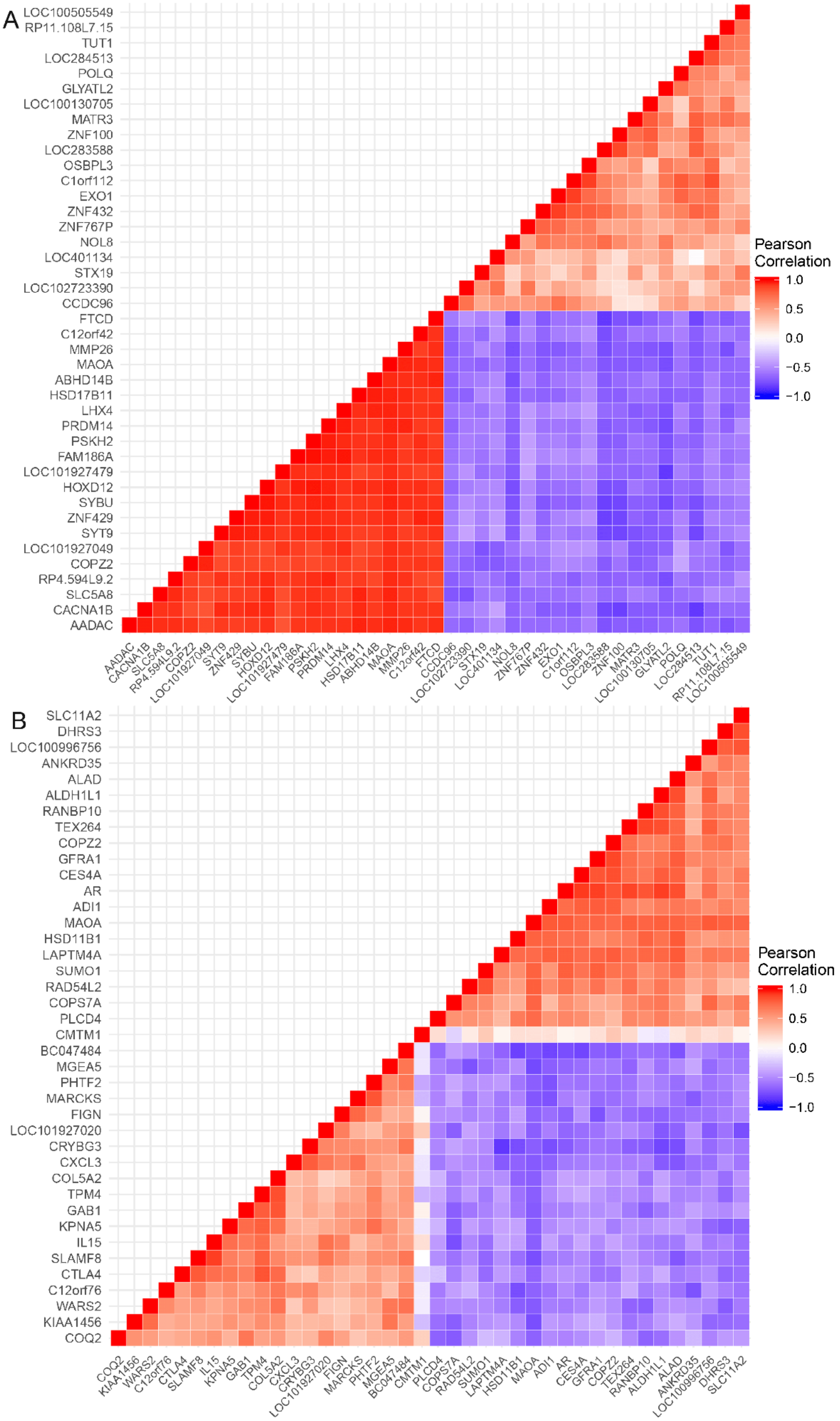

Supplementary Figure 10. Heatmaps based on the calculated Pearson's correlation coefficient of **(A)** GSE6222 and **(B)** GSE19665.

Supplementary Figure 11. Relationship network diagram based on **(A)** the positively related genes; **(B)** the negatively related genes. *PCOLCE* and *TBC1D8* were the most significant co-expressed genes positively related to *MAOA* and *PKM* were the most significant co-expressed genes negatively related to *MAOA*. The thickness of the edges was proportional to the statistical significance.

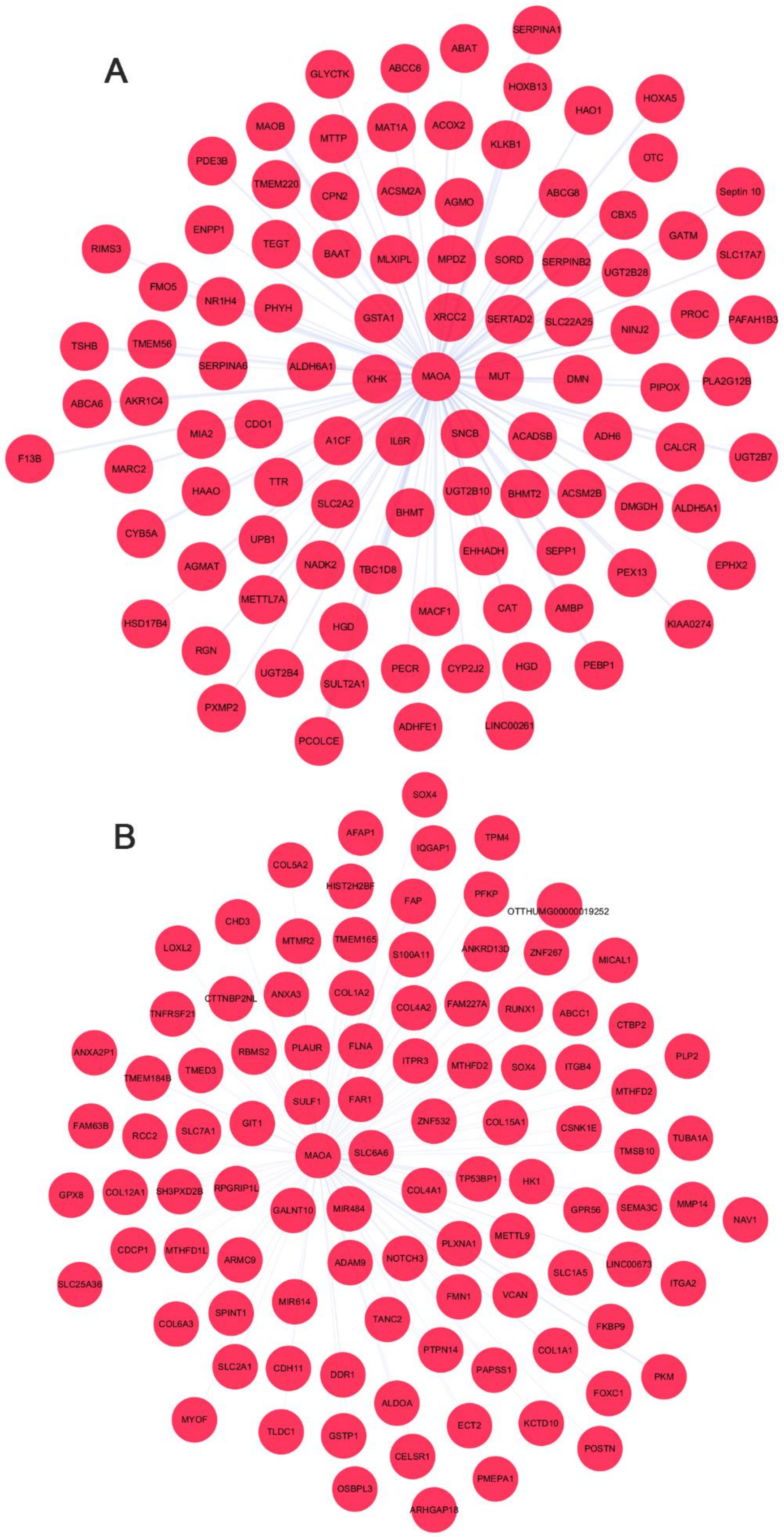

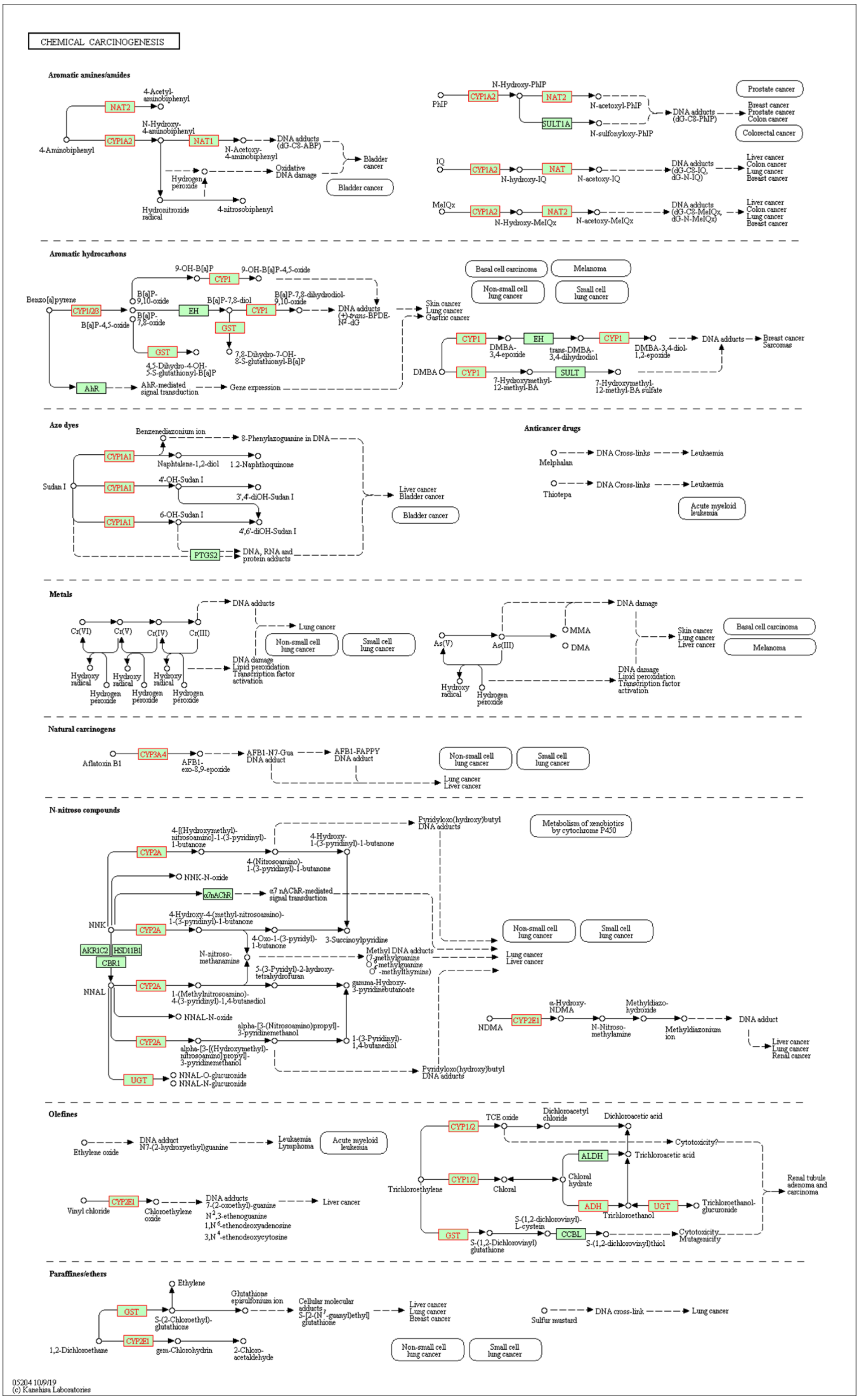

Supplementary Figure 12. The significantly clustered KEGG pathway (hsa05204) based on the intersection genes of *MAOA* positively related co-expressed genes (CEGs) and downregulated differentially expressed genes (DEGs). KEGG, the Kyoto Encyclopedia of Genes and Genomes.

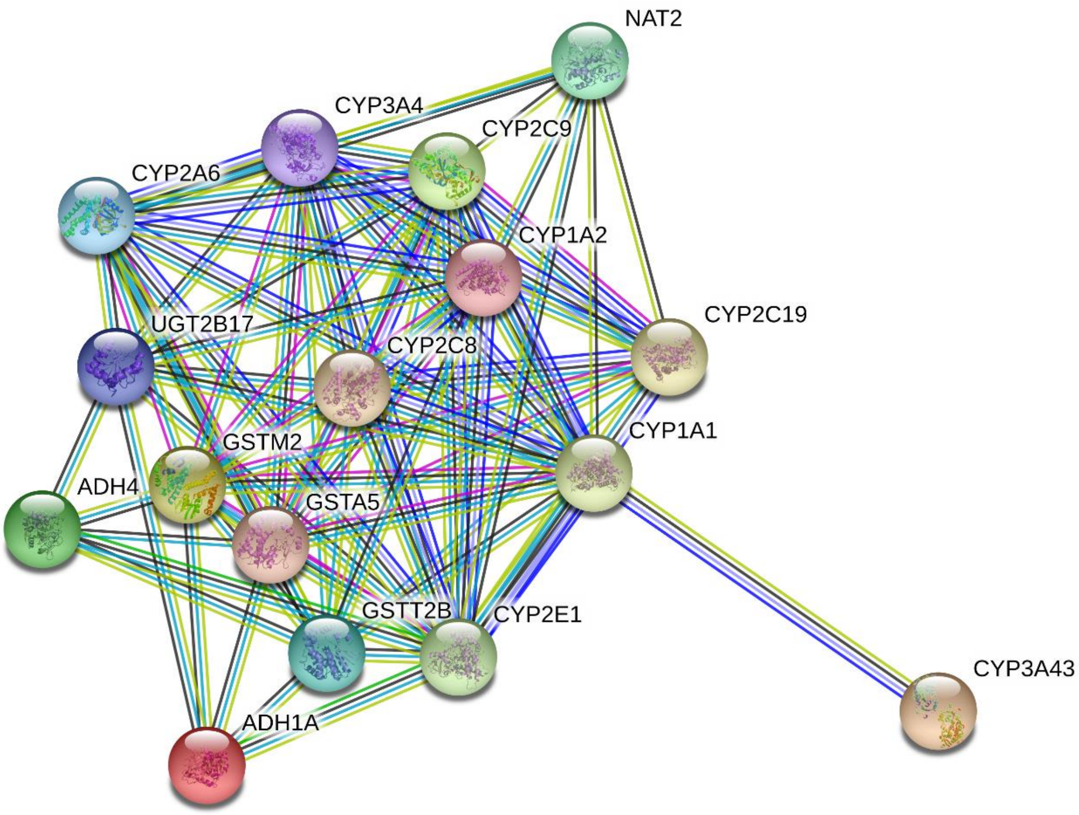

Supplementary Figure 13. Protein-to-protein interaction network based on the genes clustered in the Chemical carcinogenesis pathway.

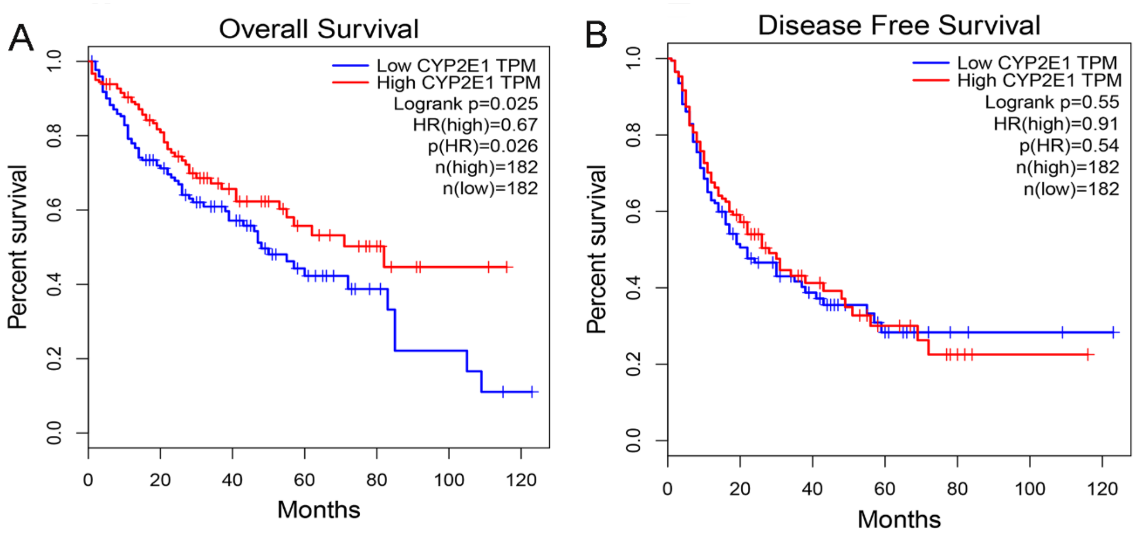

Supplementary Figure 14. Kaplan-Meier curves of the hepatocellular carcinoma patients based on the hub gene.

(A) Low CYP2E1 correlated with reduced overall survival in HCC patients. **(B)** The disease-free survival condition of low and high CYP2E1 group in HCC patients signified no significant difference.

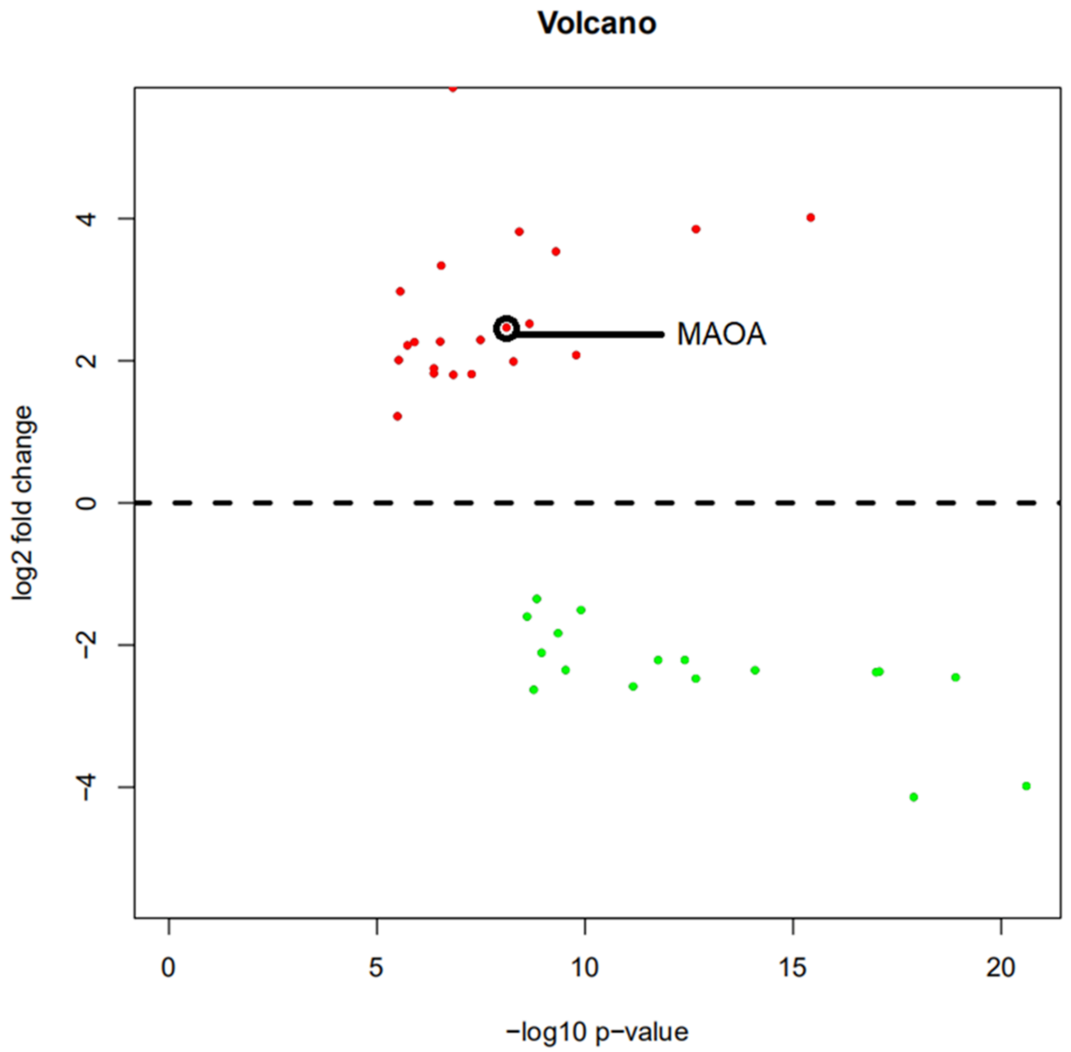

Supplementary Figure 15 A volcano plot based on differentially expressed genes of NC-treated HCC mouse model. *MAOA* expression level was significantly elevated in NC-treated group compared to control group. NC, nitidine chloride.
